# Cardiovascular risk factor mapping and distribution among adults in Mukono and Buikwe districts in Uganda: small area analysis

**DOI:** 10.1186/s12872-020-01573-3

**Published:** 2020-06-10

**Authors:** Geofrey Musinguzi, Rawlance Ndejjo, Isaac Ssinabulya, Hilde Bastiaens, Harm van Marwijk, Rhoda K. Wanyenze

**Affiliations:** 1 Department of Disease Control and Environmental Health, School of Public Health, College of Health Sciences, Makerere University, Kampala, Uganda; 2 Department of Primary and Interdisciplinary care, University of Antwerp, Antwerp, Belgium; 3 Department of Medicine, Makerere University, Kampala, Uganda; 4 Department of Primary Care and Public Health, Brighton and Sussex University Medical School, Sussex, UK

**Keywords:** Cardiovascular risk factors, Hypertension, Prevalence, Sub-Saharan Africa

## Abstract

**Background:**

Sub-Saharan Africa (SSA) is experiencing an increasing burden of Cardiovascular Diseases (CVDs). Modifiable risk factors including hypertension, diabetes, obesity, central obesity, sedentary behaviours, smoking, poor diet (characterised by inadequate vegetable and fruit consumption), and psychosocial stress are attributable to the growing burden of CVDs. Small geographical area mapping and analysis of these risk factors for CVD is lacking in most of sub-Saharan Africa and yet such data has the potential to inform monitoring and exploration of patterns of morbidity, health-care use, and mortality, as well as the epidemiology of risk factors. In the current study, we map and describe the distribution of the CVD risk factors in 20 parishes in two neighbouring districts in Uganda.

**Methods:**

A baseline survey benchmarking a type-2 hybrid stepped wedge cluster randomised trial design was conducted in December 2018 and January 2019. A sample of 4372 adults aged 25–70 years was drawn from 3689 randomly selected households across 80 villages in 20 parishes in Mukono and Buikwe districts in Uganda. Descriptive statistics and generalized linear modelling controlled for clustering were conducted for this analysis in Stata 13.0, and a visual map showing risk factor distribution developed in QGIS.

**Results:**

Mapping the prevalence of selected CVD risk factors indicated substantial gender and small area geographic heterogeneity which was masked on aggregate analysis. Patterns and clustering were observed for hypertension, physical inactivity, smoking, alcohol consumption and risk factor combination. Prevalence of unhealthy diet was very high across all parishes with no significant observable differences across areas.

**Conclusion:**

Modifiable cardiovascular risk factors are common in this low-income context. Moreover, across small area geographic setting, it appears significant differences in distribution of risk factors exist. These differences suggest that underlying drivers such as sociocultural, environmental and economic determinants may be promoting or inhibiting the observed risk factor prevalences which should be further explored. In addition, the differences emphasize the value of small geographical area mapping and analysis to inform more targeted risk reduction interventions.

## Background

More people die annually from cardiovascular diseases (CVDs) than from any other cause and three quarters of these death occur in low and middle income countries (LMICs) [1]. The high and increasing burden of CVD is attributable to a myriad of factors which include hypertension, diabetes, obesity, unhealthy diet, lack of physical activity, excessive alcohol consumption, raised blood lipids and psychosocial factors [2]. In Uganda, hypertension and other CVD risk factors are on the rise [3]; with one in four of the adult population in the district of Mukono and Buikwe reportedly hypertensive [4]. Despite the trends, data on small geographical area mapping and analysis of distribution of risk factors for CVD is lacking in most of sub-Saharan Africa and yet such data has the potential to inform monitoring of patterns of risk factors, morbidity, health-care use, mortality and the epidemiology of risk factors for more targeted risk reduction interventions [5]. In the current study, we describe the distribution of the CVD risk factors in 20 parishes in Mukono and Buikwe districts in Uganda. This research is performed within the SPICES project, an EU financed H2020 project focussing on the prevention of CVD in low and middle-income countries and vulnerable populations in Europe.

## Methods

### Purpose and specific objectives

This study was needed to inform a CVD prevention program whose aim is to improve CVD risk profiles of people in a low income setting. The specific objectives of the current study are to estimate, characterise and describe the distribution of cardiovascular risk profiles among adults in two districts of Mukono and Buikwe in Uganda.

### Design and setting of the study

**Fig. 1:**
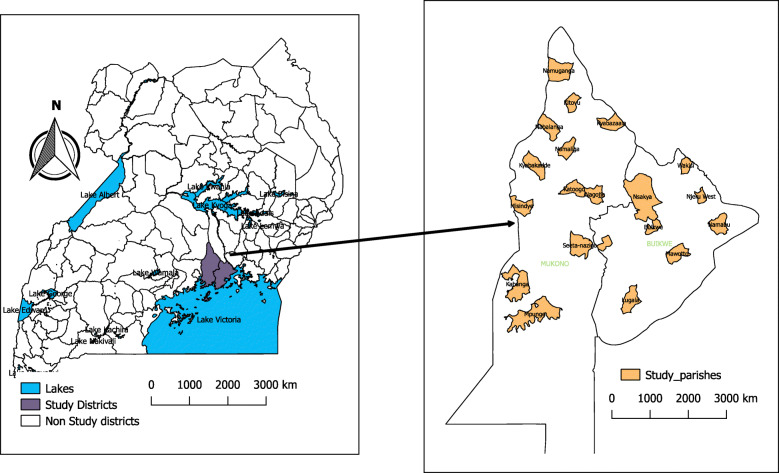
Map of Uganda and Mukono & Buikwe districts showing studied parishes

The data utilised for this risk factor mapping and analysis is drawn from the baseline survey conducted in December 2018 and January 2019 to benchmark key indicators for a type 2-hybrid stepped-wedge (SW) trial reported elsewhere [6]. The baseline was conducted in 20 parishes (Fig. 1) in the districts of Mukono (12) and Buikwe (8), home to more than 1,000,000 people. Parishes were included if they hosted a Health Centre III level facility as earlier described [6]. A health Centre III is a primary healthcare facility found at a sub-county with a general outpatient clinic and a maternity ward. Mukono and Buikwe districts are largely rural with a significant proportion (25%) living in urban or semi-urban neighbourhoods. The districts are located between the two biggest urban areas of Jinja and Kampala (the capital city).

### Characteristics of participants and sampling

A sample of 4372 adults aged 25–70 years was drawn from 3689 randomly selected households across 80 villages in 20 parishes in Mukono and Buikwe districts in Uganda. The sample size design is described elsewhere [6]. The household sample was drawn from a sampling frame generated through a mapping and listing exercise conducted prior to data collection. All households in the catchment area were each assigned a unique number. To cater for non-response, a random sample of 200 households from four villages per parish with at least one adult was generated from the original sampling frame. Sampling was conducted using the statistical software R version 3.5. The sample obtained represents an overall response rate of 92% across parishes, which is higher than the pre-determined response rate of 73.5% [6]. All samples across parishes achieved the minimum sample size requirement.

### Data collection

Study personnel (trained research assistants) approached the selected households, consented participants and proceeded with enrolment. All adults aged 25–70 years were eligible and all those who consented participated in the study. As expected, some adults were not found at home at the time of the survey. Thus, up-to three call backs were made to enhance participation. Men were more likely not to be found at home compared to women. To initiate the interview process, research assistants described the purpose of the study in the appropriate language, obtained participant consent and administered the study questionnaire following the World Health Organization (WHO) stepwise approach to chronic disease surveillance – step 1 and 2 [7]. Anthropometric and blood pressure measurements were conducted using standardised and validated equipment. Physical activity was measured using the short form of the International Physical Activity Questionnaire [8]. Diet was measured using the seven day frequency recall questionnaire – the short version of the DASH Q [9]. Blood pressure (BP) was measured on a single occasion using a validated automated digital blood pressure monitor, Omron M3 with appropriate cuff sizes. Three blood pressure measurements (at least 1 min apart) were taken after 5 min rest with the participant seated. For each participant’s blood pressure, the mean of the last two values was calculated to estimate their blood pressure.

### Data management and quality control

Research Assistants well versed with the both English and the main local language of the study (*Luganda*) received a vigorous one-week training conducted at the Makerere University School of Public Health (MakSPH) in preparation for the data collection exercise. The training entailed guided lecturers, participatory learning, role plays and pre-tests. Selected Research Assistants collected data electronically using RedCap enabled android tablets with inbuilt checks and closely supervised by the study team and a data quality supervisor and an assistant. The quality control team reviewed the electronic records to ensure that any anomalies were rectified by the data collectors while still in the field. In addition, a mid-review and additional training was conducted for two days to emphasis quality and address any critical challenges.

### Definition of variables

#### Education status

Level of education was classified into two groups. All those who had attained a level of training at a secondary or higher level were classified as post-primary, whereas those who did not receive any formal education or attained only some level of education at any primary level were classified as pre-secondary.

#### Age

Age was categorised into two groups of below 40 years and > = 40 years.

#### Smoking

Participants were considered smokers if they reported that they were currently smoking.

#### Physical inactivity

Participants were classified as physically active or inactive. Physical activity was self-reported using the validated International Physical Activity Questionnaire (IPAQ) short and scored using the MET-minutes/week (metabolic equivalent [MET]). Guidelines for Data Processing and Analysis of the International Physical Activity Questionnaire (IPAQ) were followed to conduct the analysis [10]. At least 30 min of moderate activity, (approximately 600 MET-minutes/week) were used as reference cut off to classify participants as being physically inactive or active.

#### Hypertension

A participant was classified as being hypertensive if either their mean systolic BP was 140 mmHg or higher, or average diastolic BP was 90 mmHg or higher on our measurement, or if they were on anti-hypertensive treatment in the two weeks preceding the study or a combination of the above.

#### Body Mass Index (BMI)

We weighed participants and using the WHO classification of body weight (underweight, normal weight, overweight, and obese), a cut-off of 25 was utilised to classify participants as overweight/obese if their BMI was ≥25. Pregnant women were excluded.

#### Alcohol consumption

Self-reported alcohol consumption was measured based on a 30-day recall. A recent paper published in the Lancet concluded that no level of alcohol consumption is safe [11]. Thus, we used a 30-day recall to characterise participants as alcohol consumers or not.

#### Unhealthy diet

Measured using a self-report proxy of consuming less than five servings of fruits and vegetables at least five times a week.

#### Risk factor combination

We combined four risk factors; hypertension, BMI, Alcohol consumption and Smoking to generate a composite outcome. People with 0–1 were categorised as having low risk factor combination and those with 2–4 were categorised as having high risk factor combination. This classification is based on a previously published paper in a related setting [12].

### Statistical analysis

Descriptive statistics were conducted, and a chi-square test was used to check for any differences in the distribution of risk factors across parishes. Prevalence proportion ratios with their 95% confidence intervals were computed using a generalized linear model with Poisson family, and a log link to determine the association between parish and the study outcomes. To ensure that the observed differences in prevalence are not due to confounding effects, for all outcomes, we adjusted for sex, age, religion, marital status and occupation at multivariable analysis (Table 3). All models controlled for clustering at the household level. To achieve this, we used a generalized linear model with Poisson family, and added a nonlog cluster function (household) with an eform. Analysis was carried out in STATA version 13.0 (Stata Corp, Texas, USA) statistical software. To generate the maps and to compare our sample with the 2014 Uganda National Census and Housing population, we weighted the data to the parish level by age and sex.

## Results

### Distribution of study participants by parish in Mukono and Buikwe districts

**Fig. 2:**
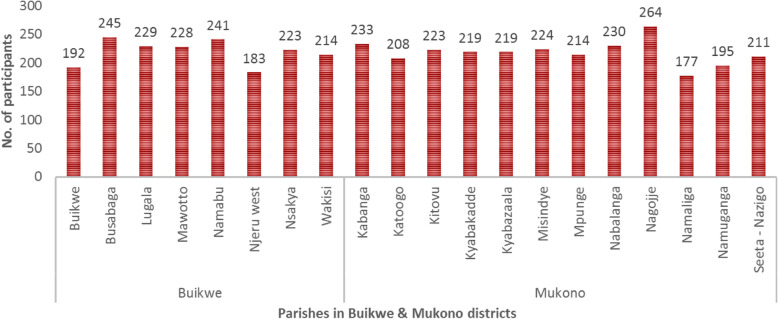
Distribution of participants by parish and district

A final sample of 4372 participants were drawn from 20 parishes into this study (Fig. 2). Mean (SD) and median participant enrolment were 218 (±20.9) and 221 respectively.

### Distribution of demographic characteristics of study participants by parish

Majority of participants (overall and across parishes) were females (60.2%), younger (55.5% were aged 25–40 years, mean age = 41.4 years (SD ± 12.74)), had obtained primary/no education (67.1%), reported doing subsistence farming or casual work (76%), were married 63.9% and catholic by religion (35.2%). Table 1 shows that across all parishes, differences in mean age, sex, education level, occupation and religion were observed; all *p*-value < 0.001).

**Table 1:** Distribution of Socio Economic and Demographic Characteristics of adult study participants by Parish, Mukono and Buikwe Districts in Uganda, *N* = 4372

Parishes	All	Buikwe	Busabaga	Kabanga	Katoogo	Kitovu	Kyabakadde	Kyabazaala	Lugala	Mawotto	Misindye	Mpunge	Nabalanga	Nagojje	Namabu	Namaliga	Namuganga	Njeru West	Nsakya	Seeta-Nazigo	Wakisi	*p*-Value
**Characteristics**																						
**Sex** Female (%)	**60.2**	69.8	61.6	56.6	61.5	62.3	58.9	59.3	53.3	69.3	67.4	63.6	57.4	48.5	53.9	72.9	49.7	72.1	52.5	57.8	63.1	< 0.001
**Age in years**																						
25–40,	**55.5**	62.5	53.1	60.1	55.3	49.8	53.9	53.9	52.8	57.0	60.3	64.5	51.7	48.9	59.8	64.4	53.3	66.1	52.0	46.0	49.5	< 0.001
41–70 (%)	**44.5**	37.5	46.9	39.9	44.71	50.2	46.12	46.1	47.2	43.0	39.7	35.5	48.3	51.1	40.3	35.6	46.7	33.9	48.0	54.0	50.5	
**Education level**																						
Primary or none	**67.1**	51.6	65.3	68.2	70.2	67.3	64.4	67.1	71.6	73.3	44.6	65.9	77.8	78.0	74.3	61.0	76.4	37.2	85.7	64.9	66.8	< 0.001
Post Primary	**32.9**	48.4	34.7	31.8	29.8	32.7	35.62	32.9	28.4	26.6	55.4	34.1	22.2	22.0	25.7	39.0	23.6	62.8	14.4	35.1	33.2	
**Occupation**																						
Farmer/casual	**76.0**	63.0	77.1	71.2	85.6	72.5	80.4	77.1	75.4	83.7	48.9	82.7	80.0	80.7	81.3	58.0	83.6	50.0	93.7	84.2	79.7	< 0.001
Informal/formal	**24.0**	37.0	22.9	28.8	14.4	27.5	19.6	22.9	24.6	16.3	51.2	17.3	20.0	19.3	18.8	42.0	16.4	50.0	6.3	15.8	20.3	
**Marital Status**																						
Never married	**8.1**	5.2	5.3	6.9	9.1	11.2	8.2	16.4	13.1	6.1	4.0	13.6	7.8	8.7	6.2	8.5	5.6	11.5	2.7	7.6	4.2	< 0.001
Married	**63.9**	63.5	65.7	66.1	63.5	62.3	63.5	55.7	59.0	62.3	66.5	58.9	60.4	63.3	73.0	60.5	65.6	71.0	73.5	62.6	60.8	
Widowed or separated	**28.0**	31.3	29.0	27.0	27.4	26.5	28.3	27.9	28.0	31.6	29.5	27.6	31.7	28.0	20.8	31.1	28.7	17.5	23.8	29.9	35.1	
**Religion**																						
Catholics	**35.2**	30.5	36.3	40.8	31.3	24.7	39.0	30.1	32.7	36.3	33.9	39.7	31.3	54.9	21.2	29.4	40.1	26.2	49.8	35.4	35.5	< 0.001
Protestant	**29.6**	25.8	30.6	24.5	28.4	35.0	24.8	22.8	43.4	35.0	39.7	30.8	37.0	28.0	13.7	24.3	31.8	25.7	27.8	40.2	21.0	
Moslems	**19.3**	26.3	12.7	15.5	22.6	26.9	15.1	23.3	8.4	12.4	11.6	11.2	20.4	6.4	51.5	32.8	9.9	32.8	13.0	4.8	34.6	
Others	**15.9**	17.4	20.4	19.3	17.8	13.5	21.1	23.7	15.5	16.4	14.7	18.2	11.3	10.6	13.7	13.6	18.2	15.3	9.4	19.6	8.9	

### Distribution of prevalence of risk factors by parish

Seven major modifiable risk factors for cardiovascular disease are included in this comparative descriptive study: – Hypertension (Table S1), high blood sugar (self-reported diabetes) (No table), overweight/obesity (Table S2), physical inactivity (Table S3), smoking (Table S4), unhealthy diet (Table S5), and alcohol consumption (Table S6).

#### Hypertension

The overall prevalence of hypertension (Table S1) in our sample was 23.4% with no overall significant difference observed among males (23.4%) compared to females (24.4%), *p* = 0.467. However, sub-analysis at the parish level revealed differences. Some parishes had higher prevalences of hypertension among men compared to females and vice versa. Higher estimates among men compared to women were observed in the parish of Nsakya, [males (29.2%) vs females (17.9%), *p* = 0.047]; Seeta-Nazigo, [males (30.3%) vs females (23.6%), *p* = 0.28] and Wakisi [males (32.9%) vs females (21.7%), *p* = 0.28]. On the other hand, higher prevalences among women compared to men were observed in the parishes of Misindye [males (19.2%) vs females 34.3%, *p* = 0.021]; and Nabalanga, [males (15.3%) vs females (26.2%), *p* = 0.04]. Meanwhile, bivariate and multivariate analysis across parishes revealed striking differences in the prevalences of hypertension, *p* = 0.001. We observed that in 11 parishes, the prevalences of hypertension were significantly different (Tables 2 and 3). The prevalence of hypertension was about twice as higher in Busabaga (adjPPR = 1.88, 95%CI: 1.27–2.77), Misindye (adjPPR = 1.98, 95%CI: 1.33–2.95), Kitovu (adjPPR = 1.86, 95%CI: 1.25–2.77), Mawoto (adjPRR = 1.80, 95%CI: 1.21–2.69) and Mpunge (adjPPR = 1.73, 95%CI: 1.14–2.63) compared to estimates in Namuganga Parish. The prevalence of hypertension was also higher in Kabanga (adjPPR = 1.75, 95%CI: 1.18–2.60), Kyabazaala (adjPPR = 1.55, 95%CI: 1.04–2.33), Lugala (adjPPR = 1.57, 95%CI: 1.05–2.34), Nsakya (adjPPR = 1.52, 95%CI: 1.00–2.31), Seeta Nazigo (adjPPR = 1.62, 95%CI: 1.07–2.45) and Wakisi (adjPPR = 1.53, 95%CI: 1.04–2.34). Meanwhile, alcohol consumption (adjPPR = 1.47, 95%CI: 1.29–1.67), higher age – 40-70 years (adjPPR = 2.34, 95%CI: 1.06–2.66), being married/cohabiting (adjPPR = 1.41, 95%CI: 1.04–1.92) and being divorced or widowed (adjPPR = 1.65, 95%CI: 1.20–2.27) also remained significantly associated with hypertension at multivariable analysis.

**Table 2:** Unadjusted associations between Parish and CVD risk Factors, SPICES Project, Mukono and Buikwe District, Uganda, N = 4372

	BP	BMI	PA	Smoking	Alcohol	Multiple
**Parish**						
Buikwe	1.18(0.75–1.85)	1.30(0.94–1.79)	3.53(0.37–33.47)	1.87(0.54–6.40)	1.08(0.63–1.84)	0.91(0.54–1.51)
Busabaga	1.98(1.33–2.93)***	1.15(0.84–1.56)	7.25(0.91–57.13)	3.29(1.05–10.25)*	2.78(1.82–4.25)***	1.95(1.29–2.94)***
Kabanga	1.66(1.11–2.47)*	1.07(0.77–1.47)	1.82(0.16–19.75)	3.65(1.19–11.19)*	2.39(1.55–3.68)***	1.47(0.96–2.25)
Katoogo	1.38(0.89–2.12)	1.08(0.78–1.50)	7.46(0.93–59.50)	2.8(0.88–8.85)	**Ref**	0.84(0.50–1.39)
Kitovu	1.89(1.27–2.80)**	1.19(0.87–1.63)	**Ref**	3.42(1.11–10.49)*	1.27(0.76–2.10)	1.24(0.78–1.94)
Kyabakadde	1.19(0.75–1.86)	1.27(0.93–1.71)	4.12(0.46–36.19)	5.32(1.76–16.02)**	2.09(1.33–3.28)***	1.44(0.92–2.26)
Kyabazaala	1.62(1.07–2.44)*	1.02(0.73–1.42)	22.48(3.07–164.67)**	2.66(0.82–8.56)	2.36(1.51–3.65)***	1.84(1.21–2.79)**
Lugala	1.59(1.06–2.38)*	1.18(0.87–1.61)	13.18(1.72–100.81)*	2.74(0.86–8.73)	2.91(1.91–4.42)***	1.50(0.97–2.30)
Mawotto	1.84(1.23–2.74)**	1.37(1.01–1.85)*	0.95(0.06–15.10)	2.95(0.94–9.16)	2.52(1.63–3.87)***	1.53(1.00–2.33)
Misindye	1.82(1.21–2.72)**	1.15(0.83–1.58)	21.79(2.98–159.34)**	**Ref**	1.71(1.06–2.75)*	1.53(0.98–2.38)
Mpunge	1.52(0.99–2.32)	1.30(0.95–1.77)	1.00(0.06–15.82)	4.19(1.37–12.79)*	1.75(1.09–2.80)*	1.19(0.75–1.91)
Nabalanga	1.33(0.86–2.04)	1.33(0.98–1.79)	18.26(2.48–134.24)**	3.31(1.07–10.16)*	1.70(1.07–2.68)*	1.37(0.89–2.12)
Nagojje	1.07(0.69–1.64)	1.35(1.01–1.81)*	3.22(0.36–28.48)	3.90(1.29–11.76) *	3.47(2.31–5.19)***	1.86(1.24–2.78)**
Namabu	1.28(0.84–1.96)	1.35(1.00–1.82)*	4.67(0.56–39.25)	3.90(1.28–11.83)*	1.31(0.80–2.14)	1.37(0.88–2.11)
**Namuganga**	**Ref**	1.37(1.00–1.86)*	2.17(0.19–23.67)	2.99(0.92–9.63)	1.75(1.09–2.78)*	**Ref**
Namaliga	1.28(0.81–2.01)	1.18(0.85–1.64)	18.32(2.45–136.78)**	4.05(1.28–12.75)*	1.65(1.00–2.69)*	1.37(0.86–2.15)
Njeru west	1.37(0.87–2.13)	1.13(0.81–1.58)	25.44(3.49–185.55)***	1.71(0.46–6.38)	1.86(1.15–3.01)*	1.43(0.90–2.26)
Nsakya	1.46(0.95–2.21)	1.28(0.94–1.74)	12.29(1.57–95.92)*	1.41(0.40–4.92)	0.93(0.54–1.59)	1.03(0.63–1.65)
Seeta - Nazigo	1.66(1.10–2.50)*	1.23(0.89–1.70)	6.09(0.71–52.13)	2.12(0.62–7.22)	1.58(0.98–2.53)	1.21(0.75–1.94)
Wakisi	1.62(1.07–2.43)*	**Ref**	6.20(0.76–50.77)	2.72(0.85–8.63)	1.48(0.91–2.38)	1.29(0.82–2.02)

*p < 0.05, **P ≤ 0.01, ***p ≤ 0.001

**Table 3:** Adjusted associations between Parish and CVD risk Factors, SPICES Project, Mukono and Buikwe District, Uganda, N = 4372

	BP	BMI	PA	Smoking	Alcohol	Multiple
**Parish**						
Buikwe	1.34 (0.85–2.11)	0.93(0.69–1.26)		1.40(0.45–4.36)	1.30(0.81–2.07)	0.99(0.80–1.21)
Busabaga	1.88 (1.27–2.77)***	0.84(0.63–1.12)		1.51(0.53–4.29)	2.43(1.70–3.46)***	1.34(1.13–1.58)***
Kabanga	1.76(1.18–2.61)**	0.77(0.57–1.04)		1.58(0.54–4.62)	2.05(1.41–2.97)***	1.22(1.02–1.44)*
Katoogo	1.48(0.96–2.26)	0.79(0.58–1.06)		1.63(0.57–4.58)	**Ref**	0.95(0.78–1.15)
Kitovu	1.87(1.25–2.78)**	0.86(0.64–1.15)		2.14(0.76–5.98)	1.28(0.83–1.97)	1.07(0.89–1.28)
Kyabakadde	1.22(0.78–1.89)	0.91(0.68–1.20)		2.69(0.96–7.50)	1.84(1.25–2.70)**	1.17(0.98–1.40)
Kyabazaala	1.56(1.04–2.33)*	0.72(0.52–0.99)*		1.24(0.42–3.68)	2.43(1.70–3.48)***	1.22(1.01–1.45)*
Lugala	1.58(1.05–2.35)*	0.88(0.66–1.17)		1.11(0.38–3.24)	2.36(1.65–3.37)***	1.24(1.04–1.46)*
Mawotto	1.81(1.21–2.70)**	1.03(0.78–1.36)		1.72(0.60–4.87)	2.31(1.61–3.33)***	1.29(1.08–1.53)**
Misindye	1.99(1.34–2.95)***	0.87(0.64–1.17)		**Ref**	1.69(1.11–2.55)*	1.29(1.08–1.56)**
Mpunge	1.74(1.14–2.64)**	0.95(0.71–1.26)		2.35(0.81–6.80)	1.55(1.03–2.33)*	1.23(1.02–1.46)*
Nabalanga	1.31(0.85–2.00)	0.97(0.73–1.28)		1.74(0.62–4.86)	1.53(1.04–2.26)*	1.11(0.93–1.32)
Nagojje	0.95(0.61–1.46)	1.01(0.77–1.31)		1.17(0.42–3.22)	2.48(1.76–3.47)***	1.23(1.04–1.45)*
Namabu	1.39(0.91–2.13)	0.96(0.72–1.26)		2.05(0.74–5.61)	1.66(1.09–2.51)*	1.03(0.85–1.24)
**Namuganga**	**Ref**	**Ref**		1.45(0.48–4.31)	1.86(1.21–2.83)**	**Ref**
Namaliga	1.25(0.77–2.02)	0.82(0.59–1.13)		3.48(1.18–10.19)*	1.51(1.01–2.24)*	1.18(0.97–1.44)
Njeru west	1.54(0.99–2.39)	0.81(0.58–1.11)		1.39(0.42–4.51)	2.51(1.62–3.85)***	1.23(1.01–1.50)*
Nsakya	1.52(1.00–2.31)*	0.94(0.71–1.25)		0.79(0.24–2.51)	0.74(0.45–1.19)	0.93(0.76–1.12)
Seeta - Nazigo	1.62(1.07–2.46)*	0.92(0.67–1.24)		1.41(0.45–4.37)	1.35(0.89–2.02)	1.02(0.84–1.24)
Wakisi	1.56(1.04–2.35)*	0.72(0.52–0.98)*		1.56(0.53–4.51)	1.46(0.97–2.19)	1.02(0.84–1.23)
**Current Smoker**						
No (ref)						
Yes	0.94(0.77–1.14)	1.10(0.92–1.33)			2.03(1.79–2.30)***	
**Alcohol**						
No (ref)						
Yes	1.47(1.29–1.68)***	0.96(0.84–1.09)		4.55(3.32–6.24)***		
**Sex**						
Female (Ref)						
Male	0.93(0.82–1.05)	1.00(0.89–1.11)		4.18(3.11–5.61)***	1.83(1.63–2.04)***	1.38(1.30–1.46)***
**Age**						
25- < 40 (Ref)						
Age (40–70)	2.35(2.06–2.67)***	0.93(0.83–1.03)		1.43(1.11–1.84)**	1.09(0.97–1.22)	1.35(1.27–1.44)***
**Education** (post Primary)	0.98(0.86–1.11)	0.94(0.84–1.04)		0.63(0.47–0.83)***	0.86(0.76–0.98)*	0.86(0.80–0.92)***
**BMI**						
< 25 year (ref)						
≥ 25				1.11(0.88–1.38)	0.97(0.86–1.08)	
**Blood Pressure**						
Normal (Ref)						
High		1.00(0.89–1.13)		0.98(0.77–1.24)	1.38(1.23–1.54)***	
**Religion**						
Catholic (Ref)						
Protestant	0.99(0.87–1.13)	0.96(0.85–1.09)		0.72(0.55–0.94)*	0.81(0.72–0.90)***	
Muslim	1.08(0.91–1.27)	1.06(0.92–1.22)		1.72(1.20–2.47)**	0.19(0.14–0.26)***	
Other	0.89(0.74–1.06)	1.08(0.93–1.24)		0.67(0.39–1.14)	0.19(0.14–0.26)***	
**Marital status**						
Single (Ref)						
Cohabiting or married	1.42(1.04–1.92)*	1.06(0.88–1.27)		0.67(0.46–0.97)*	1.09(0.90–1.33)	1.09(0.96–1.23)
Divorced or widowed	1.65(1.20–2.27)**	1.10(0.89–1.35)		1.34(0.89–2.00)	1.05(0.85–1.31)	1.23(1.07–1.40)**
**Occupation**						
Sub-Farming (ref)						
Informal/formal	1.01(0.88–1.16)	1.06(0.94–1.19)		0.62(0.44–0.87)**		1.03(0.96–1.11)

*p < 0.05, **P < 0.01, ***p < 0.001

#### Self-reported diabetes

The prevalences of diabetes was reportedly 1.6% overall, lower among males (1.1%) compared to females (1.9%), *p* = 0.029. Further analysis by parish level was not conducted due to inadequate sample sizes. Only those who reported having ever been screened for blood sugar were asked their diabetes status.

#### Overweight/obesity

The overall prevalence of overweight/obesity (Table S2) in our sample was 30.4% with no overall significant difference observed among males (30.2%) compared to females (30.6%). Further analysis at bivariate revealed significant prevalence proportion ratios in the parishes of Mawotto (unadjPPR = 1.37, 95%CI: 1.01–1.85), Nagojje (unadjPPR = 1.35, 95%CI: 1.01–1.81), Namabu (adjPPR = 1.35, 95%CI: 1.00–1.82) and Namuganga (adjPPR = 1.37, 95%CI: 1.00–1.86), (Table 2). The associations were not retained at multivariate analysis, (Table 3).

#### Physical inactivity

The overall prevalence of physical inactivity was 4.0%, [lower among males (3.0%) compared to females (4.8%)], *p* = 0.002. By parish, differences in physical inactivity were also observed, *p* < 0.001. Indeed, physical inactivity was highest in Njeru West (12%), [higher among males (12.7%) compared to females (11.7%)]; and lowest in Mpunge and Mawotto parishes at (0.5%), with no observed difference by gender. Overall, all parishes had lower levels of physical inactivity. At bivariate analysis, differences were observed for 6 of the parishes. However, the confidence intervals were very wide possibly due to very small numbers in the comparison as majority of the people fell within the physically active category (Table 2). Thus, we never carried forward to analyse the outcomes at multivariate to avoid very small cells.

#### Smoking

The overall prevalence of smoking was 6.8%, [higher among males (13%) than females (2.7%)], *p* < 0.001. Differences across parishes were prevalent *p* = 0.012. Smoking prevalences were relatively higher in Kyabakadde (11.9%), [higher among males (17.7%) than females (7.7%), *p* = 0.024]; Namaliga (9.0%), [higher among males (22.9%) than females (3.8%), *p* < 0.001]; and Mpunge (9.3%), [lower among males (8.7%) thanfemales (9.8%), *p* = 0.008]. Lowest smoking prevalences were noted in Buikwe parish (4.1%), [higher among males (8.6%) thanfemales (2.2%)], *p* = 0.04, Njeru West (3.8%), [higher among males (9.8%) thano females (1.5%)], *p* = 0.009; and Nsakya (3.1%), [only among males (6.6%)] and Misindye (3.2%), [only among males (7.2%)]. At bivariate and multivariate analysis, differences were only retained in Namaliga parish (adjPPR = 3.47, 95%CI: 1.18–10.19). This possibly meant parish was not a predictor but rather, other attributes may explain the smoking behaviour. Indeed, alcohol consumption (adjPPR = 4.55, 95%CI: 3.32–6.24), male gender (adjPPR = 4.18, 95%CI: 3.11–5.60), higher age (adjPPR = 1.43, 95%CI: 1.11–1.84), and Muslim religion (adjPPR = 1.72, 95%CI: 1.20–2.47) remained significantly associated with smoking practices at multivariate analysis. Moreover, post primary education (adjPPR = 0.62, 95%CI: 0.47–0.82), protestant religion (adjPPR = 0.72, 95%CI: 0.55–0.93), cohabiting/being married (adjPPR = 0.66, 95%CI: 0.46–0.96) and formal/ informal occupation other than subsistence farming (adjPPR = 0.62, 95%CI: 0.44–0.86) had lower prevalence proportional ratios, suggesting that these are possibly protective factors against smoking.

#### Alcohol consumption

The overall prevalence of alcohol consumption was 23.0%, significantly higher among males (34.6%) than females (15.5%), *p* < 0.001 and across parishes, *p* < 0.001. By parish, highest prevalences were observed in Nagojje (41.7%), [higher among males, (58.8%) compared to females (23.4%), *p* < 0.001]; Lugala, 38%, [higher among males (47.7%) compared to females (24.0%), *p* < 0.001]; and Busabaga (33.5%), [higher among males (49.0%) than females (23.8%), *p* < 0.001]. Lowest prevalences of alcohol consumption were observed in Nsakya (11.2%), [higher among males (17.0%) thanfemales (6.0%)]; Buikwe, (13.0%), [higher among males (22.4%) than females (9.0%)]; and Namabu (15.7%), [higher among males 19.8% thanfemales (12.3%)]. At bivariate and multivariate analysis, significant differences were retained across 14 parishes (Tables 2 and 3). In addition, high blood pressure (adjPPR = 1.37, 95%CI: 1.23–1.53), smoking (adjPPR = 2.03, 95%CI: 1.79–2.04), and male gender (adjPPR = 1.82, 95%CI: 1.63–2.04) remained significant with higher prevalence proportional ratios at multivariate analysis. On the other hand, being protestant (adjPPR = 0.80, 95%CI: 0.72–0.99), Muslim (adjPPR = 0.19, 95%CI: 0.14–0.25), and other religion (adjPPR = 0.19, 95%CI: 0.14–0.26) retained low prevalence proportion ratios compared to being catholic at multivariate analysis.

#### Unhealthy diet

Inadequate fruit and vegetable consumption was used as a proxy measure for unhealthy diet. The prevalence of unhealthy diet was (88.6%), with no significant differences among males (88.7%) compared to females (88.5%), *p* = 0.831. Across all parishes, inadequate fruit and vegetable consumption was very prevalent and we opted not to conduct further analysis.

#### Risk factor combination

We combined four risk factors; hypertension, BMI, alcohol consumption and smoking to generate a composite outcome (Tables 2 and 3). People with high risks (≥ 2 risk factors) were more in Busabaga (adjPPR = 1.33, 95%CI: 1.13–1.58), Kabanga (adjPPR = 1.21, 95%CI: 1.02–1.43), Kyabazaala (adjPPR = 1.21, 95%CI: 1.01–1.45), Lugaala (adjPPR = 1.23, 95%CI: 1.04–1.46), Mawoota (adjPPR = 1.28, 95%CI: 1.08–1.52), Misindye (adjPPR = 1.29, 95%CI: 1.07–1.55), Mpunge (adjPPR = 1.22, 95%CI: 1.02–1.46), Nagojj (adjPPR = 1.22, 95%CI: 1.04–1.44), and Njeru West (adjPPR = 1.23, 95%CI: 1.01–1.50). Other independent predictors of high risk category were being male (adjPPR = 1.37, 95%CI: 1.30–1.45), older age (40–70) (adjPPR = 1.35, 95%CI: 1.27–1.43) and being divorced or widowed (adjPPR = 1.22, 95%CI: 1.07–1.40). Post primary education had a protective association (adjPPR = 0.85, 95%CI: 0.80–0.91).

#### Patterns and risk factor mapping

To generate a visual and illustrative picture of the patterns and cluster distribution of risk factors across parishes, we weighted the data to the 2014 Uganda National Housing and Census population for the individual parishes and colour coded the distribution based on descriptive data. Using QGIS, distribution densities represented by colour codes were overlaid on the parish maps of Mukono and Buikwe (Figure supplement 1) and a colour code table (Table 4) generated. It is interesting to note that when the data was aggregated (overall), variations were masked and yet specific parish colour coding show differences in risk factors and lifestyles. Commensurate with the descriptive statistics, differences are evident in the distribution of CVD risk factors in the various parishes.

**Table 4:**
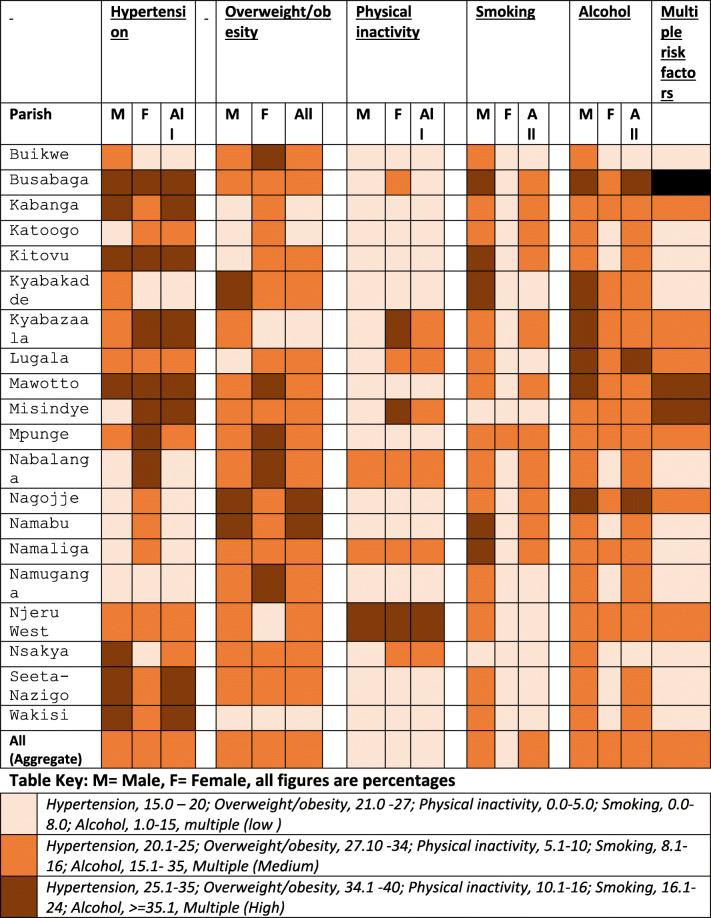
Parish and sex-specific colour coded prevalences of weighted risks factors -- A Cardiovascular Disease Risk Factor Atlas among adults in Mukono and Buikwe districts in Uganda – Analysis of Baseline data: The SPICES Project

## Discussion

This study included a mapping and analysis of the distribution of CVD risk factors in 20 parishes in two neighbouring districts in Uganda. The study highlights new and important findings on the distribution of cardiovascular risk factors across smaller geographical areas in a low-income context. Consistent with other findings in the country [13] and around the region [14], aggregate prevalences of risk factors for CVDs are generally high except for smoking at 6.7% and physical inactivity at 4.1%. The prevalence of hypertension in both districts stands at 23.4%, with no significant difference across gender and the prevalence of overweight/obesity is 30.4% with no observed difference as well by gender. Other prevalent risk factors are unhealthy diet (88.8%), no difference by gender; and alcohol consumption (23.0%) higher among males. Less prevalent risk factors are physical inactivity and self-reported diabetes.

These results are important and support previous evidence on the prevalence of CVD risk factors [4, 13–15]. But more importantly, further analysis of the data at the granular or small geographical area, by parish, gender, age and at multivariate level reveal even more intriguing findings regarding the distribution of CVD risk factors. The data shows that across small area geographical spans, several differences in the distribution of risk factors exist. For example, the prevalence of hypertension is almost as twice as higher in some parishes compared to others. Likewise, analysis by gender at parish level reveal differences. Whereas most parishes have higher prevalences of hypertension among men (1.5 times higher or more in some parishes) than women, other parishes have quite way-higher prevalences of hypertension among females than males. For example, in Misindye parish, the estimated prevalence of hypertension is 1.66 times higher among females (34.3%) compared to males (19.2%). Incidentally, known risk factors except physical inactivity which is higher among females (12.9%) compared to males (4.5%), don’t seem to be different. In fact, most of the risk factors are more prevalent among males than females. Again, smoking is noticeably higher among males than females in all parishes except for Mpunge Parish. These findings reveal that CVD risk factors in this setting may be distributed in a clustered pattern. The drivers for the observed patterns are not clear but social-economic and cultural environment, access to health services and local cultural practices might be responsible. For instance, Misindye parish is largely a densely populated urban setting with an array of small shops and local markets which are mainly run by females. Nsakya also has two local markets that mainly drive the local economy. Boda boda (commercial motorcycles) as a means of low-cost local transportation is booming and is quickly replacing other means of transport such as cycling and walking to work. These practices are possibly driving the sedentary behaviours observed in these settings especially among females. In Mpunge parish, the high rates of smoking among females appear to be driven by pipe smoking – a behaviour attributed to underlying cultural and social drivers. This atlas of CVD risk factors distribution in Mukono and Buikwe in Uganda suggests existence of geographic patterning and distribution of CVD risk factors. For example, marked patterning is visible regarding the prevalence of hypertension, alcoholism, and smoking. Very limited patterning is noticeable regarding dietary consumptions of fruits and vegetables. Meanwhile, smoking prevalence is low overall except for a few pockets and this is consistent with a previous study in the country [16].

Limitations: Self-reported estimates of risk behaviours in this analysis might be underestimated due to recall bias. Fruit and vegetable consumption may follow a sessional pattern; thus a longitudinal observation of dietary behaviours may provide more concrete data on practices. The sample size may be considered fairly small. Generalizability of the study findings should be applied with caution given the clustering effect and the fact that more women than men were surveyed. Moreover, the areas compared are not of equal sizes and may not be homogeneous. Nevertheless, these findings are insightful and call for an in-depth exploration of the drivers that could explain these trends. For example, exploring socio-economic, psychosocial and environmental determinants may provide explanations for the observed patterning. The high prevalence of risk behaviours calls for innovate strategies to reverse the trends. This Atlas is useful and serves as a baseline for the analysis of trends and has potential to guide intervention programmes. Beyond the study districts, the findings of this study should trigger interest in similar low income settings to explore CVD risk factor heterogeneity to inform targeted interventions. Moreover, it might stimulate studies in the country and elsewhere to characterise and map the distribution of CVD risk factors on both large and small scales in order to inform global, national and local level programing and responses.

## Conclusion

Modifiable cardiovascular risk factors are prevalent in this low-income context. Moreover, across small area geographic settings, significant differences in distribution of risk factors exist. These differences suggest that underlying drivers such as sociocultural determinants may be promoting or inhibiting the observed risk factor prevalences. These drivers may warrant exploration to understand observed patterns and strategies are needed to mitigate the risk factors to minimise morbidity and mortality due to CVD endpoints.

## Supplementary information

**Additional file 1: Table S1**. Parish and sex-specific prevalence hypertension -- A Cardiovascular Disease Risk Factor Atlas among adults in Mukono and Buikwe districts in Uganda – Analysis of Baseline data: The SPICES Project.

**Additional file 2: Table S2**. Parish and sex-specific prevalence Overweight/Obesity -- A Cardiovascular Disease Risk Factor Atlas among adults in Mukono and Buikwe districts in Uganda – Analysis of Baseline data: The SPICES Project.

**Additional file 3: Table S3**. Parish and sex-specific prevalence Physical Inactivity -- A Cardiovascular Disease Risk Factor Atlas among adults in Mukono and Buikwe districts in Uganda – Analysis of Baseline data: The SPICES Project.

**Additional file 4: Table S4**. Parish and sex-specific prevalence of Smoking -- A Cardiovascular Disease Risk Factor Atlas among adults in Mukono and Buikwe districts in Uganda – Analysis of Baseline data: The SPICES Project.

**Additional file 5: Table S5**. Parish and sex-specific prevalence of alcohol consumption -- A Cardiovascular Disease Risk Factor Atlas among adults in Mukono and Buikwe districts in Uganda – Analysis of Baseline data: The SPICES Project.

**Additional file 6: Table S6**. Parish and sex-specific prevalence of fruits and vegetables consumption -- A Cardiovascular Disease Risk Factor Atlas among adults in Mukono and Buikwe districts in Uganda – Analysis of Baseline data: The SPICES Project.

**Additional file 7: Figure S1**. Map of Muono and Buikwe district showing the distribution of various Cardivacular Disease Risk Factors.

## Data Availability

All data presented in the current study are available from the corresponding author on reasonable request.

## Abbreviations

BMI: Body mass index
CVD: Cardiovascular disease
DASHQ: Dietary approach to stop hypertension questionnaire
IPAQ: International physical activity questionnaire
LMIC: Low and middle income country
NCD: Non communicable disease
