# Supplementary material for: Cardiovascular risk factor mapping and distribution among adults in Mukono and Buikwe districts in Uganda: small area analysis
Source: BMC Cardiovasc Disord. 2020 Jun 10;20:284. doi: 10.1186/s12872-020-01573-3 (PMC7288476; doi:10.1186/s12872-020-01573-3)
Supplement: Supplementary file 1 — Additional file 1: Table S1. Parish and sex-specific prevalence hypertension -- A Cardiovascular Disease Risk Factor Atlas among adults in Mukono and Buikwe districts in Uganda – Analysis of Baseline data: The SPICES Project. [file 12872_2020_1573_MOESM1_ESM.docx]

**TABLE S1. Parish and sex-specific prevalence hypertension -- A Cardiovascular Disease Risk Factor Atlas among adults in Mukono and Buikwe districts in Uganda – Analysis of Baseline data: The SPICES Project**

| **Prevalence of hypertension** | | | | | | |
| --- | --- | --- | --- | --- | --- | --- |
|  | **Un-weighted data** | | | **Weighted data** | | |
| Parish | Men (%) | Women (%) | Overall (%) | Men (%) | Women (%) | Overall (%) |
| Buikwe | 22.4 | 17.5 | 19.0 | 22.6 | 17.3 | 19.6 |
| Busabaga | 34.0 | 30.2 | 31.8 | 34.2 | 29.9 | 32.2 |
| Kabanga | 30.7 | 23.4 | 26.6 | 31.2 | 23.2 | 27.7 |
| Katoogo | 17.5 | 25.2 | 22.2 | 17.6 | 24.8 | 21.1 |
| Kitovu | 32.1 | 29.1 | 30.3 | 32.5 | 28.9 | 30.8 |
| Kyabakadde | 21.1 | 17.6 | 19.1 | 21.4 | 17.4 | 19.6 |
| Kyabazaala | 23.6 | 27.8 | 26.1 | 24.2 | 27.4 | 25.7 |
| Lugala | 24.3 | 26.7 | 25.6 | 24.5 | 24.5 | 25.3 |
| Mawotto | 27.1 | 30.8 | 29.6 | 30.4 | 27.4 | 29.1 |
| Misindye | 19.2 | 34.3 | 29.2 | 19.5 | 33.9 | 27.4 |
| Mpunge | 21.8 | 26.0 | 24.4 | 21.9 | 25.7 | 23.8 |
| Nabalanga | 15.3 | 26.2 | 21.4 | 15.7 | 25.9 | 20.1 |
| Nagojje | 12.5 | 22.3 | 17.1 | 12.7 | 22.1 | 16.0 |
| Namabu | 18.0 | 23.1 | 20.6 | 18.1 | 22.8 | 19.9 |
| Namaliga | 18.8 | 21.4 | 20.6 | 19.0 | 21.2 | 20.3 |
| Namuganga | 19.4 | 12.6 | 16.1 | 19.6 | 12.5 | 16.9 |
| Njeru West | 19.6 | 23.0 | 22.0 | 20.0 | 22.7 | 21.6 |
| Nsakya | 29.2 | 17.9 | 23.4 | 29.5 | 17.7 | 24.9 |
| Seeta-Nazigo | 30.3 | 23.6 | 26.7 | 30.4 | 23.4 | 27.5 |
| Wakisi | 32.9 | 21.7 | 26.0 | 33.3 | 21.4 | 27.3 |
| **All** | **23.4** | **24.4** | **23.4** | **23.7** | **24.1** | **23.9** |
