# Supplementary material for: Cardiovascular risk factor mapping and distribution among adults in Mukono and Buikwe districts in Uganda: small area analysis
Source: BMC Cardiovasc Disord. 2020 Jun 10;20:284. doi: 10.1186/s12872-020-01573-3 (PMC7288476; doi:10.1186/s12872-020-01573-3)
Supplement: Supplementary file 2 — Additional file 2: Table S2. Parish and sex-specific prevalence Overweight/Obesity -- A Cardiovascular Disease Risk Factor Atlas among adults in Mukono and Buikwe districts in Uganda – Analysis of Baseline data: The SPICES Project. [file 12872_2020_1573_MOESM2_ESM.docx]

**TABLE S2. Parish and sex-specific prevalence Overweight/Obesity -- A Cardiovascular Disease Risk Factor Atlas among adults in Mukono and Buikwe districts in Uganda – Analysis of Baseline data: The SPICES Project**

| **Prevalence of Overweight/Obesity** | | | | | | |
| --- | --- | --- | --- | --- | --- | --- |
|  | **Un-weighted data** | | | **Weighted data** | | |
| Parish | Men (%) | Women (%) | Overall (%) | Men (%) | Women (%) | Overall (%) |
| Buikwe | 27.6 | 34.9 | 32.6 | 27.6 | 34.9 | 31.8 |
| Busabaga | 28.7 | 28.8 | 28.7 | 28.8 | 28.8 | 28.8 |
| Kabanga | 25.7 | 27.3 | 26.6 | 25.7 | 27.4 | 26.4 |
| Katoogo | 25.0 | 28.5 | 27.1 | 25.0 | 28.4 | 26.6 |
| Kitovu | 26.2 | 32.3 | 29.9 | 26.0 | 32.4 | 29.0 |
| Kyabakadde | 37.8 | 27.2 | 31.6 | 37.6 | 27.1 | 32.8 |
| Kyabazaala | 29.2 | 23.0 | 25.6 | 29.3 | 22.9 | 26.4 |
| Lugala | 26.2 | 32.8 | 29.6 | 26.1 | 32.9 | 28.8 |
| Mawotto | 30.0 | 36.4 | 34.3 | 29.9 | 36.4 | 33.5 |
| Misindye | 28.8 | 28.8 | 28.8 | 28.7 | 28.7 | 28.8 |
| Mpunge | 26.9 | 35.9 | 32.5 | 27.1 | 35.9 | 31.6 |
| Nabalanga | 31.6 | 34.4 | 33.2 | 31.7 | 34.5 | 32.9 |
| Nagojje | 38.2 | 28.9 | 33.9 | 38.4 | 28.9 | 35.0 |
| Namabu | 36.9 | 30.8 | 33.8 | 37.1 | 30.8 | 34.6 |
| Namaliga | 31.3 | 29.1 | 29.7 | 30.8 | 29.2 | 29.8 |
| Namuganga | 31.6 | 36.8 | 34.2 | 31.7 | 36.8 | 33.6 |
| Njeru West | 33.3 | 26.2 | 28.3 | 33.3 | 26.2 | 29.1 |
| Nsakya | 30.2 | 33.9 | 32.1 | 30.3 | 34.1 | 31.8 |
| Seeta-Nazigo | 29.2 | 32.1 | 30.7 | 29.1 | 32.1 | 30.3 |
| Wakisi | 24.1 | 25.6 | 25.0 | 24.0 | 25.6 | 24.8 |
| **All** | 30.2 | 30.6 | 30.4 | 30.2 | 30.6 | 30.4 |
