# Supplementary material for: Cardiovascular risk factor mapping and distribution among adults in Mukono and Buikwe districts in Uganda: small area analysis
Source: BMC Cardiovasc Disord. 2020 Jun 10;20:284. doi: 10.1186/s12872-020-01573-3 (PMC7288476; doi:10.1186/s12872-020-01573-3)
Supplement: Supplementary file 3 — Additional file 3: Table S3. Parish and sex-specific prevalence Physical Inactivity -- A Cardiovascular Disease Risk Factor Atlas among adults in Mukono and Buikwe districts in Uganda – Analysis of Baseline data: The SPICES Project. [file 12872_2020_1573_MOESM3_ESM.docx]

**TABLE S3. Parish and sex-specific prevalence Physical Inactivity -- A Cardiovascular Disease Risk Factor Atlas among adults in Mukono and Buikwe districts in Uganda – Analysis of Baseline data: The SPICES Project**

| **Prevalence of Physical inactivity** | | | | | | |
| --- | --- | --- | --- | --- | --- | --- |
|  | **Un-weighted data** | | | **Weighted data** | | |
| Parish | Men (%) | Women (%) | Overall (%) | Men (%) | Women (%) | Overall (%) |
| Buikwe | 3.8 | 0.8 | 1.7 | 3.9 | 0.8 | 2.0 |
| Busabaga | 0.0 | 5.5 | 3.4 | 0.0 | 5.5 | 2.8 |
| Kabanga | 0.0 | 1.5 | 0.9 | 0.0 | 1.5 | 0.7 |
| Katoogo | 1.3 | 4.9 | 3.5 | 1.4 | 4.9 | 3.1 |
| Kitovu | 0.0 | 0.8 | 0.5 | 0.0 | 0.7 | 0.4 |
| Kyabakadde | 2.5 | 1.6 | 1.9 | 2.5 | 1.6 | 2.1 |
| Kyabazaala | 4.6 | 15.3 | 10.6 | 4.6 | 15.4 | 9.3 |
| Lugala | 4.0 | 8.6 | 6.2 | 4.0 | 8.6 | 5.7 |
| Mawotto | 1.4 | 0.0 | 0.5 | 1.5 | 0.0 | 0.6 |
| Misindye | 4.5 | 12.9 | 10.3 | 4.6 | 13.0 | 9.5 |
| Mpunge | 0.0 | 0.7 | 0.5 | 0.0 | 0.7 | 0.4 |
| Nabalanga | 7.8 | 9.2 | 8.6 | 7.8 | 9.2 | 8.4 |
| Nagojje | 0.7 | 2.3 | 1.5 | 0.8 | 2.3 | 1.3 |
| Namabu | 2.0 | 2.4 | 2.2 | 2.3 | 2.0 | 2.2 |
| Namaliga | 9.1 | 8.5 | 8.6 | 8.9 | 8.5 | 8.7 |
| Namuganga | 1.0 | 1.0 | 1.0 | 1.1 | 1.0 | 1.0 |
| Njeru West | 12.8 | 11.7 | 12.0 | 13.0 | 11.5 | 12.1 |
| Nsakya | 4.1 | 7.7 | 5.8 | 4.1 | 7.8 | 5.4 |
| Seeta-Nazigo | 3.4 | 2.5 | 2.9 | 3.4 | 2.5 | 3.0 |
| Wakisi | 4.1 | 2.3 | 2.9 | 4.1 | 2.3 | 3.1 |
| **All** | **2.9** | **4.8** | **4.1** | **2.9** | **4.8** | **3.8** |
