# Supplementary material for: Cardiovascular risk factor mapping and distribution among adults in Mukono and Buikwe districts in Uganda: small area analysis
Source: BMC Cardiovasc Disord. 2020 Jun 10;20:284. doi: 10.1186/s12872-020-01573-3 (PMC7288476; doi:10.1186/s12872-020-01573-3)
Supplement: Supplementary file 4 — Additional file 4: Table S4. Parish and sex-specific prevalence of Smoking -- A Cardiovascular Disease Risk Factor Atlas among adults in Mukono and Buikwe districts in Uganda – Analysis of Baseline data: The SPICES Project. [file 12872_2020_1573_MOESM4_ESM.docx]

**TABLE S4. Parish and sex-specific prevalence of Smoking -- A Cardiovascular Disease Risk Factor Atlas among adults in Mukono and Buikwe districts in Uganda – Analysis of Baseline data: The SPICES Project**

| **Prevalence of Smoking** | | | | | | |
| --- | --- | --- | --- | --- | --- | --- |
|  | **Un-weighted data** | | | **Weighted data** | | |
| Parish | Men (%) | Women (%) | Overall (%) | Men (%) | Women (%) | Overall (%) |
| Buikwe | 8.6 | 2.2 | 4.2 | 8.7 | 2.2 | 4.9 |
| Busabaga | 17.0 | 1.3 | 7.4 | 17.1 | 1.3 | 9.3 |
| Kabanga | 11.9 | 5.3 | 8.2 | 12.0 | 5.4 | 9.0 |
| Katoogo | 15.0 | 0.8 | 6.3 | 15.1 | 0.8 | 8.0 |
| Kitovu | 17.9 | 1.4 | 7.6 | 18.1 | 1.4 | 9.7 |
| Kyabakadde | 17.8 | 7.8 | 11.9 | 18.0 | 7.7 | 13.2 |
| Kyabazaala | 11.2 | 2.3 | 5.9 | 11.5 | 2.3 | 7.1 |
| Lugala | 12.1 | 0.8 | 6.1 | 12.3 | 0.8 | 7.6 |
| Mawotto | 15.7 | 2.5 | 6.6 | 15.9 | 2.5 | 8.1 |
| Misindye | 6.8 | 0.0 | 2.2 | 7.1 | 0.0 | 3.1 |
| Mpunge | 9.0 | 9.6 | 9.3 | 9.1 | 9.6 | 9.4 |
| Nabalanga | 13.3 | 3.0 | 7.4 | 13.4 | 3.0 | 8.7 |
| Nagojje | 14.7 | 2.3 | 8.7 | 15.0 | 2.3 | 10.4 |
| Namabu | 18.0 | 0.8 | 8.7 | 18.4 | 0.8 | 11.0 |
| Namaliga | 22.9 | 3.9 | 9.0 | 23.3 | 3.8 | 11.1 |
| Namuganga | 8.2 | 5.2 | 6.7 | 8.3 | 5.1 | 7.1 |
| Njeru West | 9.8 | 1.5 | 3.8 | 9.8 | 1.5 | 4.7 |
| Nsakya | 6.6 | 0.0 | 3.1 | 6.7 | 0.0 | 4.0 |
| Seeta-Nazigo | 10.1 | 0.8 | 4.7 | 10.1 | 0.8 | 5.9 |
| Wakisi | 11.4 | 3.0 | 6.1 | 11.4 | 3.0 | 7.1 |
| **All** | **12.9** | **2.7** | **6.8** | **13.0** | **2.7** | **8.0** |
