# Supplementary material for: Cardiovascular risk factor mapping and distribution among adults in Mukono and Buikwe districts in Uganda: small area analysis
Source: BMC Cardiovasc Disord. 2020 Jun 10;20:284. doi: 10.1186/s12872-020-01573-3 (PMC7288476; doi:10.1186/s12872-020-01573-3)
Supplement: Supplementary file 5 — Additional file 5: Table S5. Parish and sex-specific prevalence of alcohol consumption -- A Cardiovascular Disease Risk Factor Atlas among adults in Mukono and Buikwe districts in Uganda – Analysis of Baseline data: The SPICES Project. [file 12872_2020_1573_MOESM5_ESM.docx]

**TABLE S5. Parish and sex-specific prevalence of alcohol consumption -- A Cardiovascular Disease Risk Factor Atlas among adults in Mukono and Buikwe districts in Uganda – Analysis of Baseline data: The SPICES Project**

| **Prevalence of Alcoholism** | | | | | | |
| --- | --- | --- | --- | --- | --- | --- |
|  | **Un-weighted data** | | | **Weighted data** | | |
| Parish | Men (%) | Women (%) | Overall (%) | Men (%) | Women (%) | Overall (%) |
| Buikwe | 22.4 | 9.0 | 13.0 | 22.4 | 8.9 | 14.5 |
| Busabaga | 48.9 | 23.8 | 33.5 | 49.0 | 23.9 | 36.5 |
| Kabanga | 32.7 | 25.8 | 28.8 | 32.9 | 25.9 | 29.7 |
| Katoogo | 28.8 | 1.6 | 12.0 | 29.1 | 1.6 | 15.4 |
| Kitovu | 26.2 | 8.6 | 15.3 | 26.4 | 8.6 | 17.4 |
| Kyabakadde | 40.0 | 14.7 | 25.1 | 40.3 | 14.8 | 28.3 |
| Kyabazaala | 42.7 | 18.5 | 28.3 | 43.2 | 18.3 | 31.5 |
| Lugala | 47.7 | 23.8 | 34.9 | 47.9 | 23.7 | 37.9 |
| Mawotto | 48.6 | 22.2 | 30.3 | 48.5 | 22.2 | 33.2 |
| Misindye | 27.4 | 17.2 | 20.5 | 27.5 | 17.2 | 21.8 |
| Mpunge | 30.8 | 15.4 | 21.0 | 31.0 | 15.5 | 23.0 |
| Nabalanga | 27.6 | 15.2 | 20.4 | 27.8 | 15.1 | 22.1 |
| Nagojje | 58.8 | 23.4 | 41.7 | 59.3 | 23.3 | 46.2 |
| Namabu | 19.8 | 12.3 | 15.8 | 20.1 | 12.2 | 16.8 |
| Namaliga | 29.2 | 16.3 | 19.8 | 29.6 | 16.3 | 21.3 |
| Namuganga | 32.7 | 9.3 | 21.0 | 33.0 | 9.2 | 24.0 |
| Njeru West | 31.4 | 18.9 | 22.4 | 31.8 | 19.0 | 23.9 |
| Nsakya | 17.0 | 6.0 | 11.2 | 17.0 | 6.0 | 12.6 |
| Seeta-Nazigo | 29.2 | 11.5 | 19.0 | 29.4 | 11.5 | 21.3 |
| Wakisi | 29.1 | 11.1 | 17.8 | 29.4 | 11.1 | 20.1 |
| **All** | **34.4** | **15.5** | **23.0** | **34.6** | **15.5** | **25.4** |
