# Supplementary material for: Cardiovascular risk factor mapping and distribution among adults in Mukono and Buikwe districts in Uganda: small area analysis
Source: BMC Cardiovasc Disord. 2020 Jun 10;20:284. doi: 10.1186/s12872-020-01573-3 (PMC7288476; doi:10.1186/s12872-020-01573-3)
Supplement: Supplementary file 6 — Additional file 6: Table S6. Parish and sex-specific prevalence of fruits and vegetables consumption -- A Cardiovascular Disease Risk Factor Atlas among adults in Mukono and Buikwe districts in Uganda – Analysis of Baseline data: The SPICES Project. [file 12872_2020_1573_MOESM6_ESM.docx]

**TABLE S6. Parish and sex-specific prevalence of fruits and vegetables consumption -- A Cardiovascular Disease Risk Factor Atlas among adults in Mukono and Buikwe districts in Uganda – Analysis of Baseline data: The SPICES Project**

| 5 or more servings of Fruits and Vegetables | | | | | | |
| --- | --- | --- | --- | --- | --- | --- |
| Unweighted data | | | | Weighted data | | |
| Parish (%) | Men (%) | Women (%) | Overall (%) | Men (%) | Women (%) | Overall (%) |
| Buikwe | 98.3 | 100 | 99.5 | 98.3 | 100 | 99.3 |
| Busabaga | 61.7 | 64.9 | 63.7 | 61.7 | 65.0 | 63.3 |
| Kabanga | 95.0 | 94.7 | 94.9 | 94.9 | 94.7 | 94.8 |
| Katoogo | 98.7 | 96.1 | 97.1 | 98.8 | 96.1 | 97.5 |
| Kitovu | 100 | 97.1 | 98.2 | 100 | 97.1 | 98.6 |
| Kyabakadde | 46.7 | 54.3 | 51.1 | 46.7 | 54.2 | 50.2 |
| Kyabazaala | 100 | 99.2 | 99.5 | 100 | 99.2 | 99.6 |
| Lugala | 95.3 | 94.3 | 94.8 | 95.3 | 94.3 | 94.9 |
| Mawotto | 81.4 | 82.3 | 82.0 | 81.2 | 82.4 | 81.9 |
| Misindye | 97.3 | 91.4 | 93.3 | 97.2 | 91.4 | 94.0 |
| Mpunge | 100 | 97.1 | 98.1 | 100 | 97.0 | 98.5 |
| Nabalanga | 99.0 | 99.2 | 99.1 | 99.0 | 99.2 | 99.1 |
| Nagojje | 98.5 | 97.7 | 98.1 | 98.5 | 97.7 | 98.2 |
| Namabu | 60.4 | 56.2 | 58.1 | 60.2 | 56.2 | 58.5 |
| Namaliga | 93.7 | 92.2 | 92.7 | 93.8 | 92.2 | 92.8 |
| Namuganga | 99.0 | 97.9 | 98.5 | 99.0 | 98.0 | 98.6 |
| Njeru West | 98.0 | 95.5 | 96.2 | 98.0 | 95.5 | 96.4 |
| Nsakya | 97.2 | 98.3 | 97.8 | 97.2 | 98.3 | 97.7 |
| Seeta-Nazigo | 74.2 | 77.1 | 75.8 | 74.1 | 76.9 | 75.4 |
| Wakisi | 91.1 | 91.1 | 91.1 | 91.2 | 91.0 | 91.1 |
| All Parishes | 88.7 | 88.5 | 88.6 | 88.7 | 88.5 | 88.6 |
