# Supplementary material for: Cardiovascular risk factor mapping and distribution among adults in Mukono and Buikwe districts in Uganda: small area analysis
Source: BMC Cardiovasc Disord. 2020 Jun 10;20:284. doi: 10.1186/s12872-020-01573-3 (PMC7288476; doi:10.1186/s12872-020-01573-3)
Supplement: Supplementary file 7 — Additional file 7: Figure S1. Map of Muono and Buikwe district showing the distribution of various Cardivacular Disease Risk Factors. [file 12872_2020_1573_MOESM7_ESM.pdf]

Map of Mukon and Buikwe districts showing the overall weighted prevalence of hypertension by parish

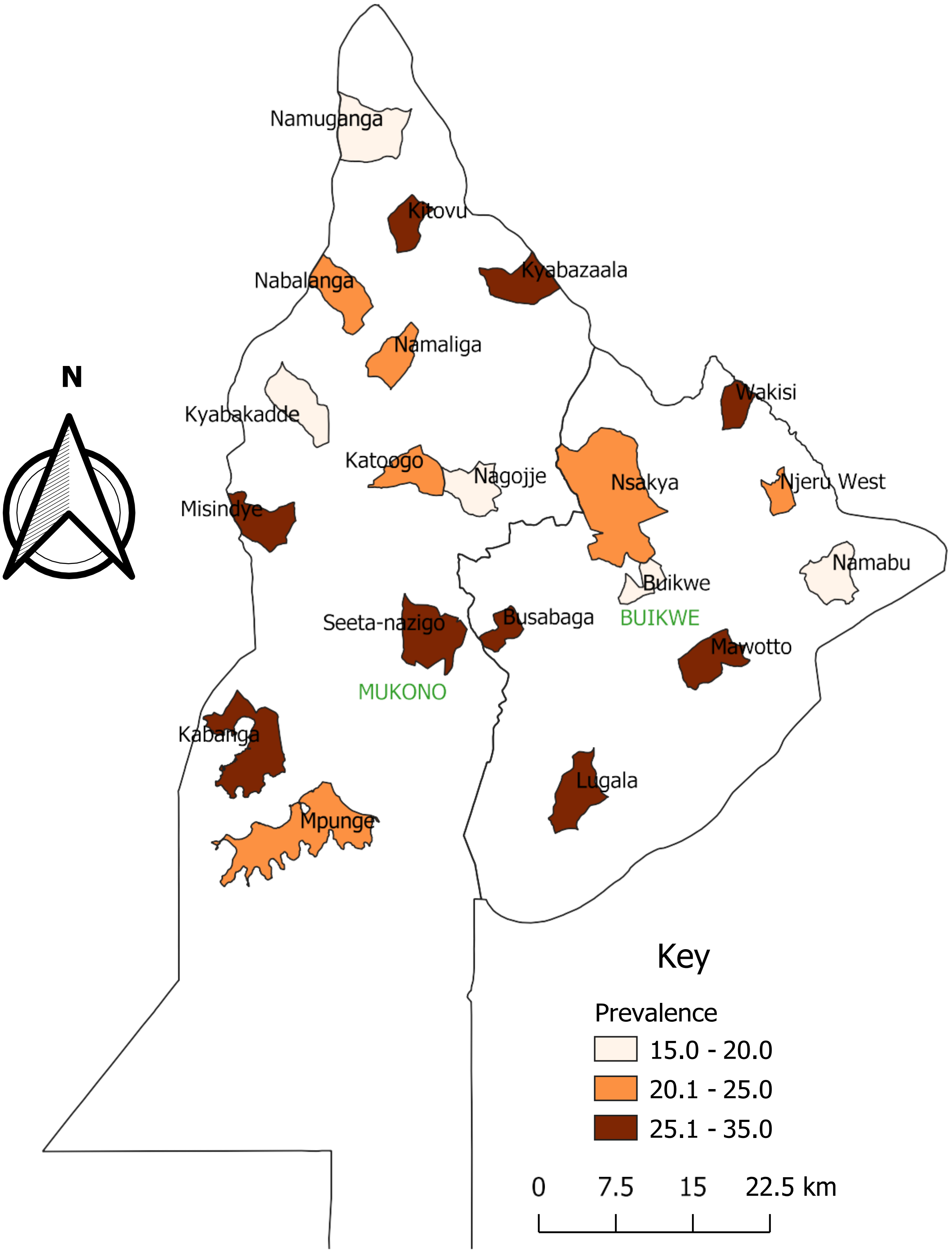

Map of Mukono and Buikwe districts showing the weighted prevalence of hypertension among men by parish

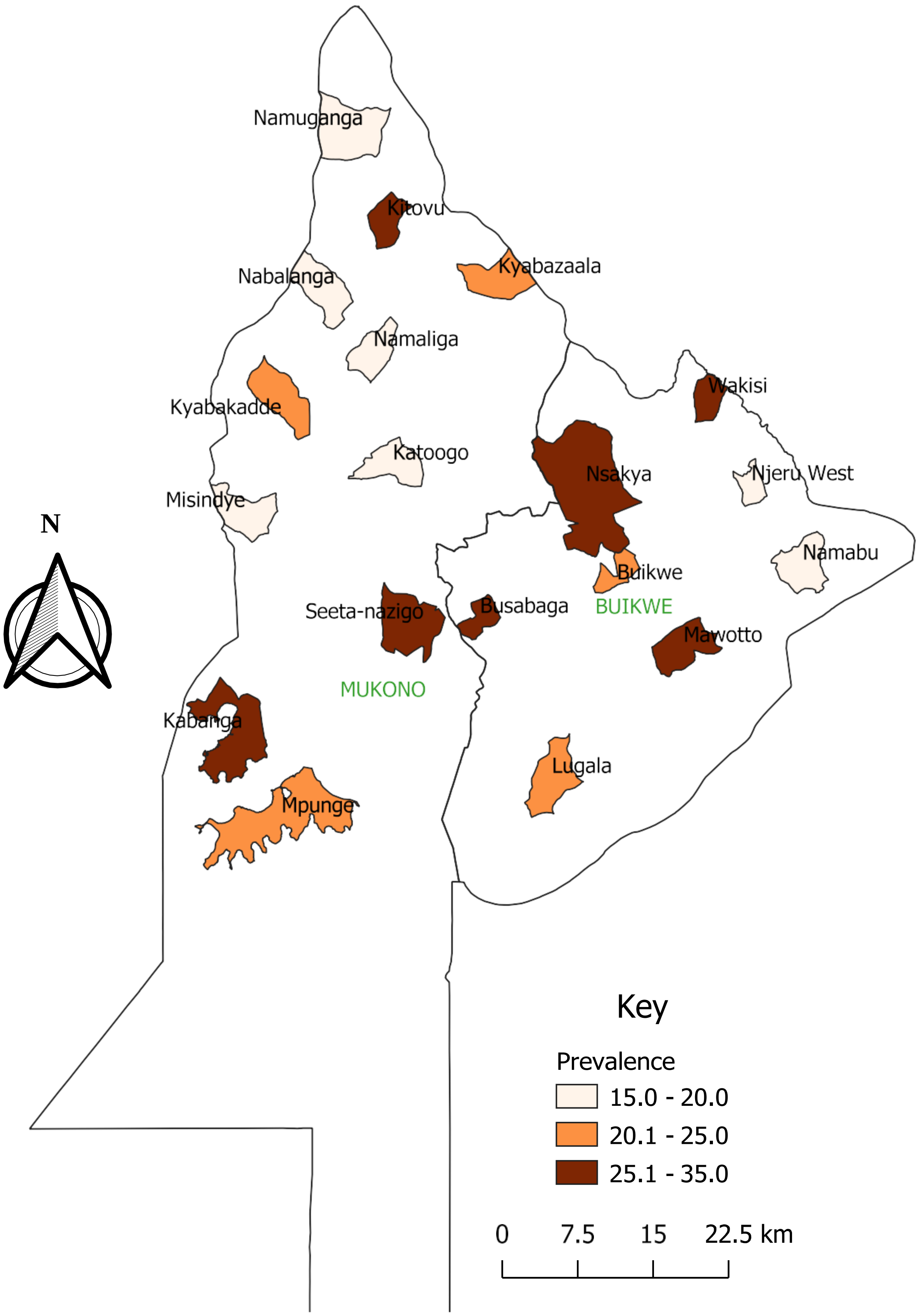

Map of Mukono and Buikwe districts showing the weighted prevalence of hypertension among women

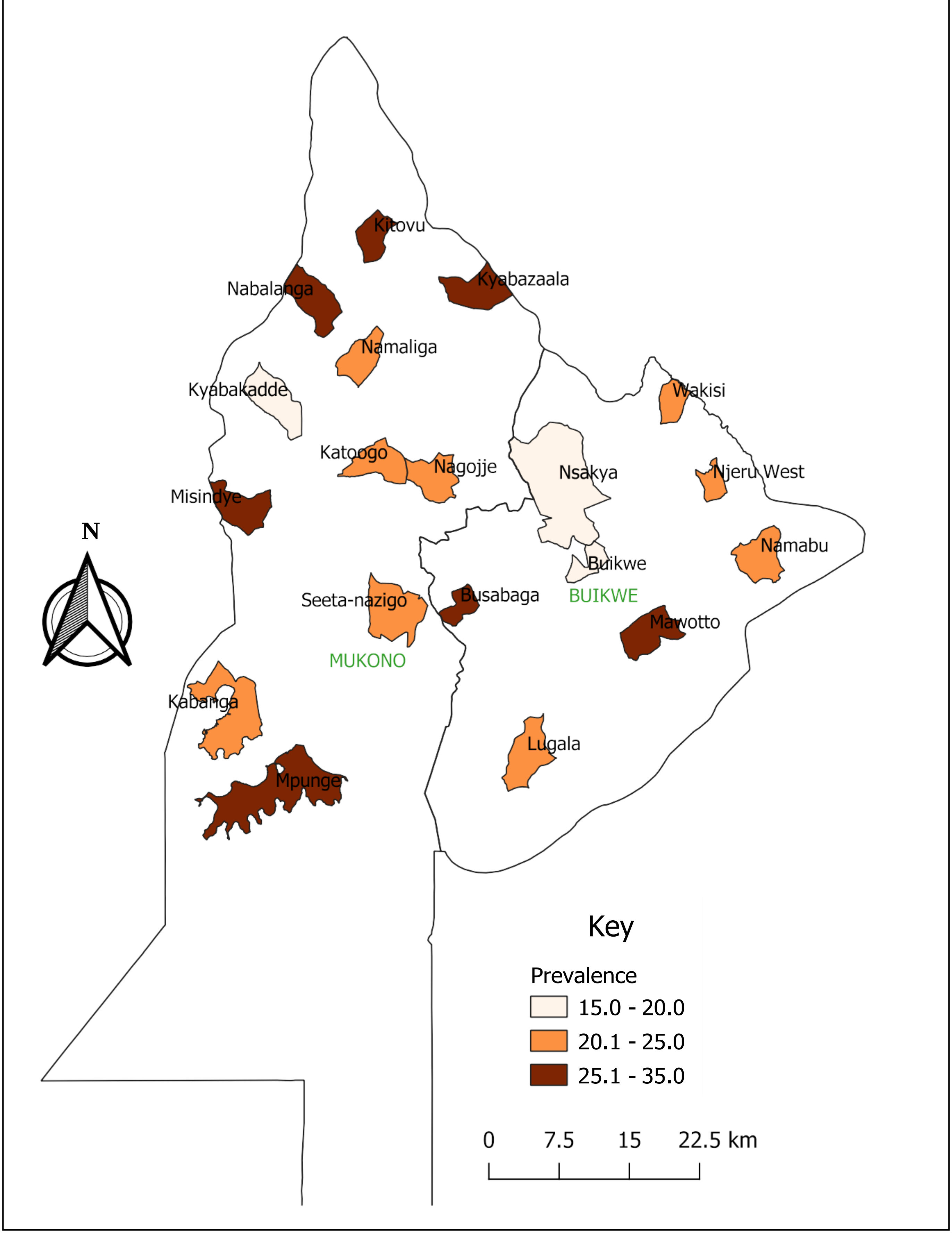

Map of Mukono and Bbuikwe districts showing the overall weighted prevalence of obesity/ overweight by parish

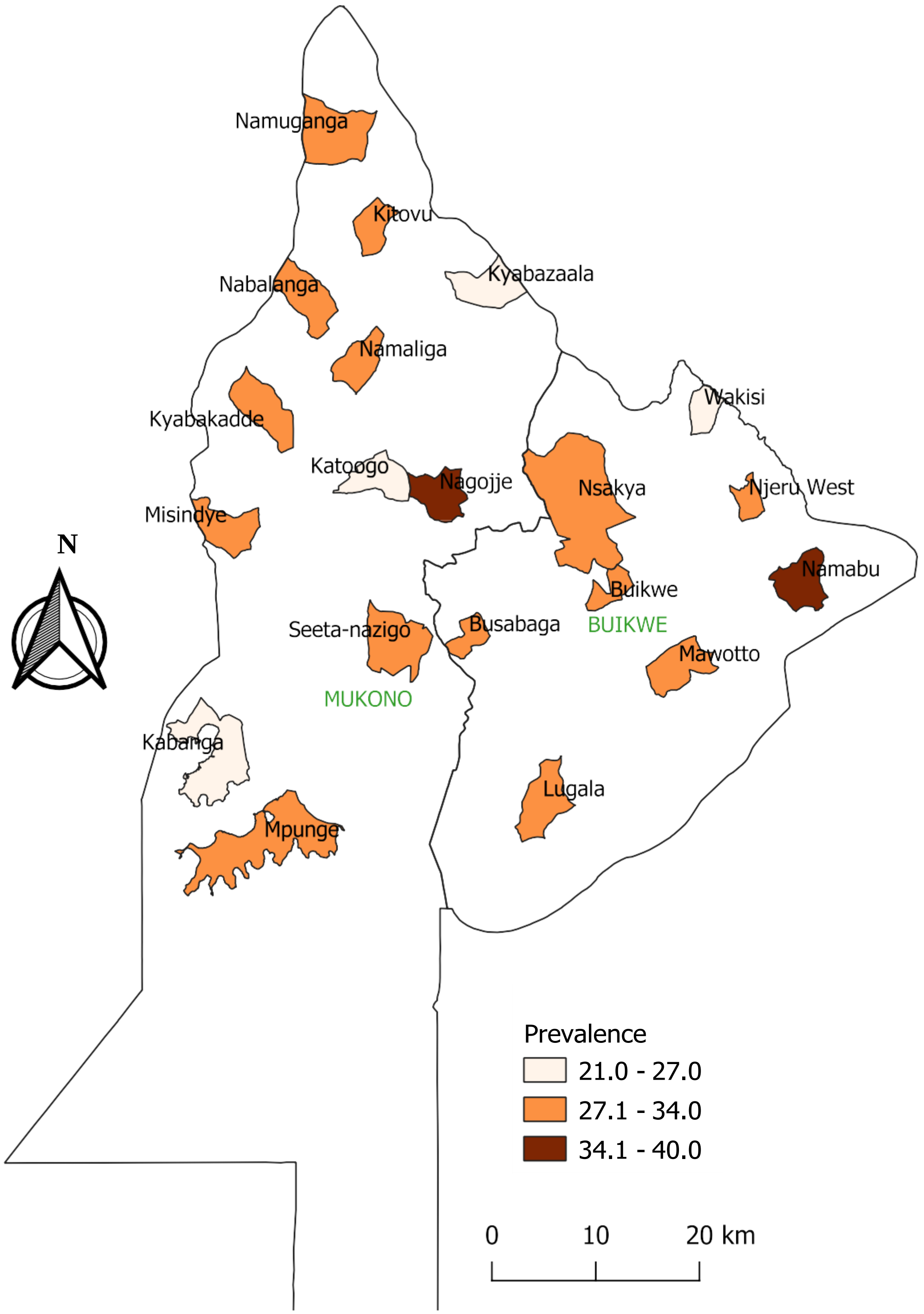

Map of Mukono and Buikwe disitricts showing the weighted prevalence of obesity/ overweight among men

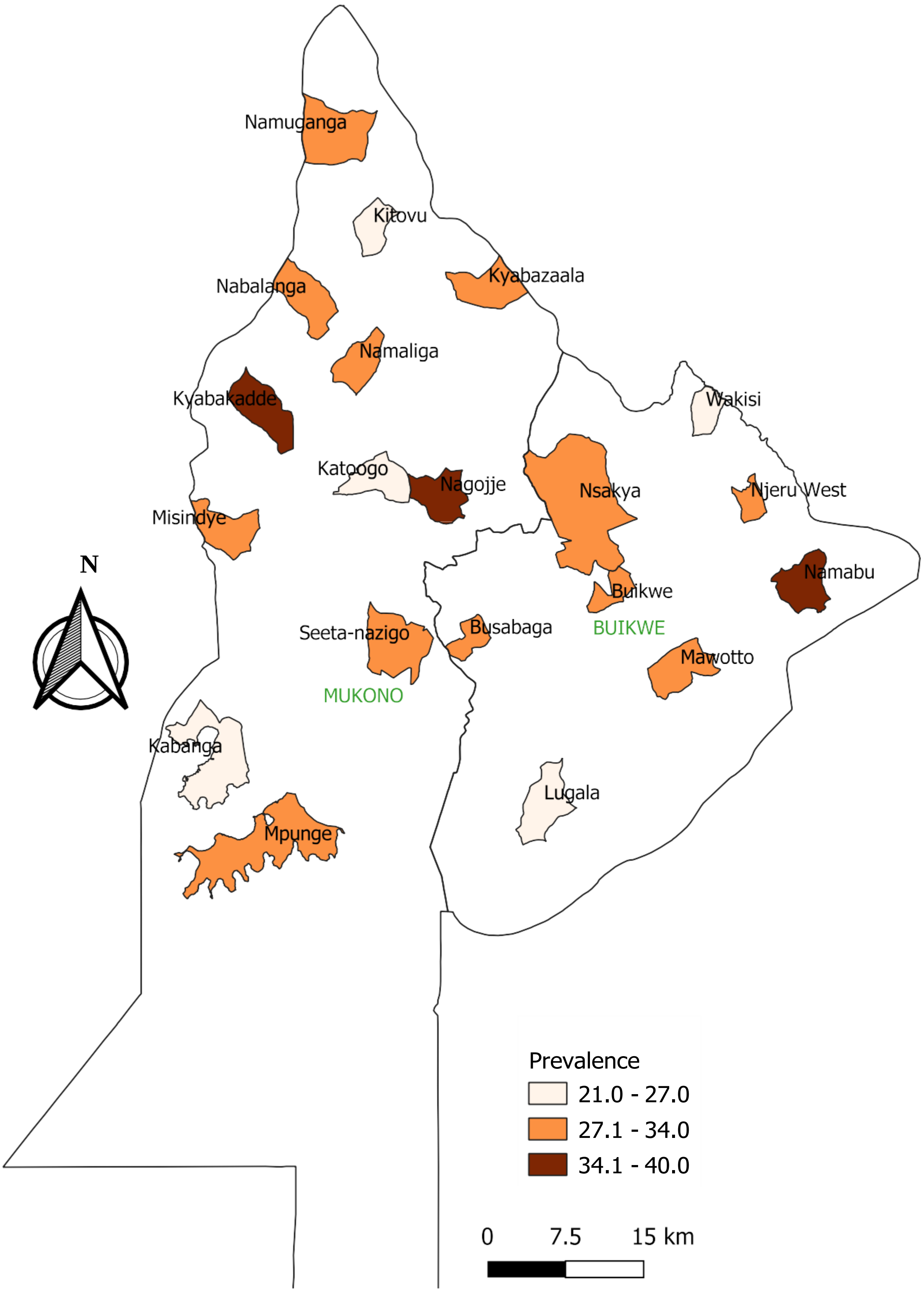

Map of Mukono and Buikwe districts showing the prevalence of obesity/ over weight among women by parish

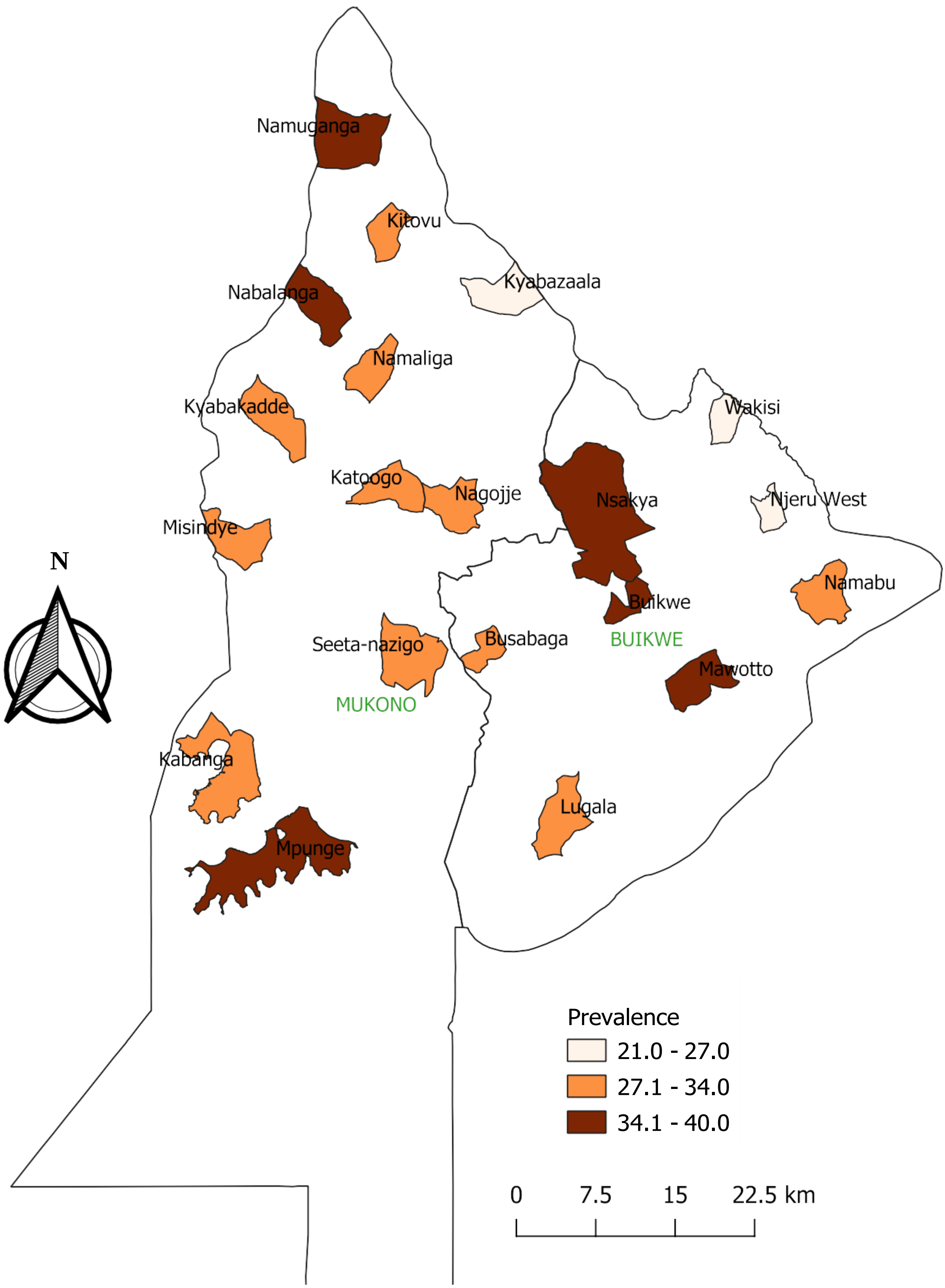

Map of Mukono and Buikwe districts showing the overall weighted prevalence of physical inactivity

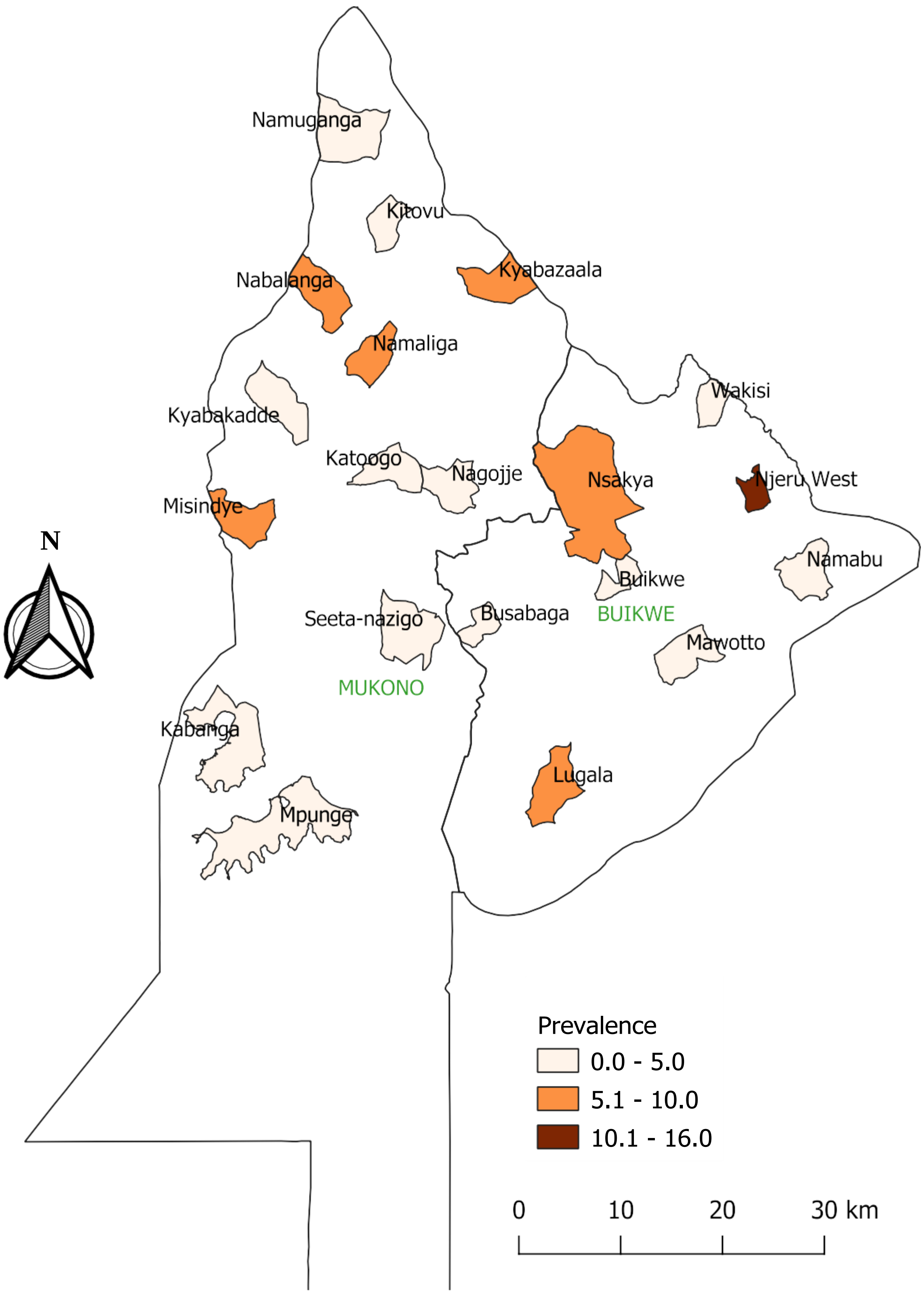

Map of Mukono and Buikwe districts showing the weighted prevalence of physical inactivity among men by parish

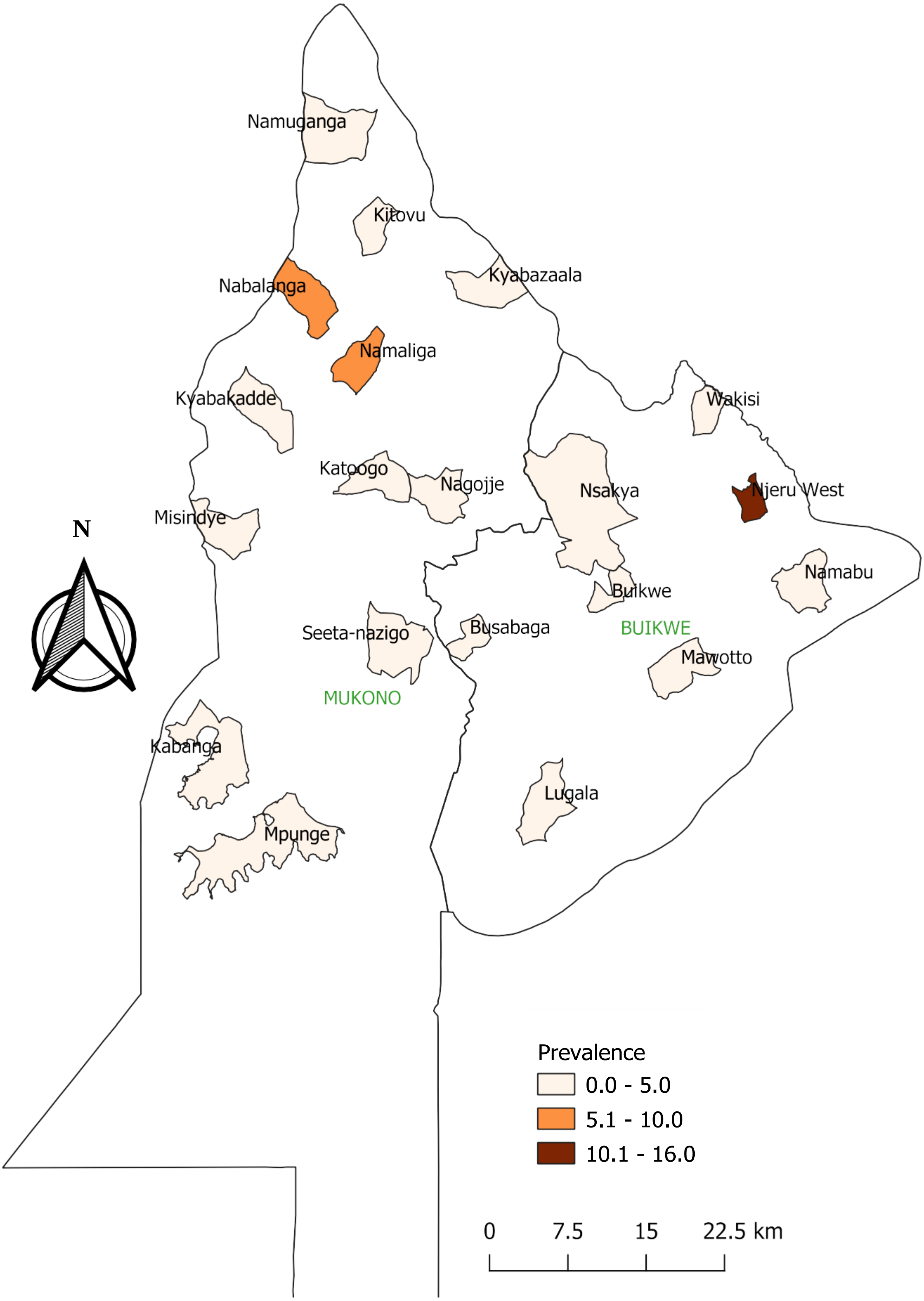

Map of Mukono and Buikwe districts showing the prevalence of physical inactivity among women by parish

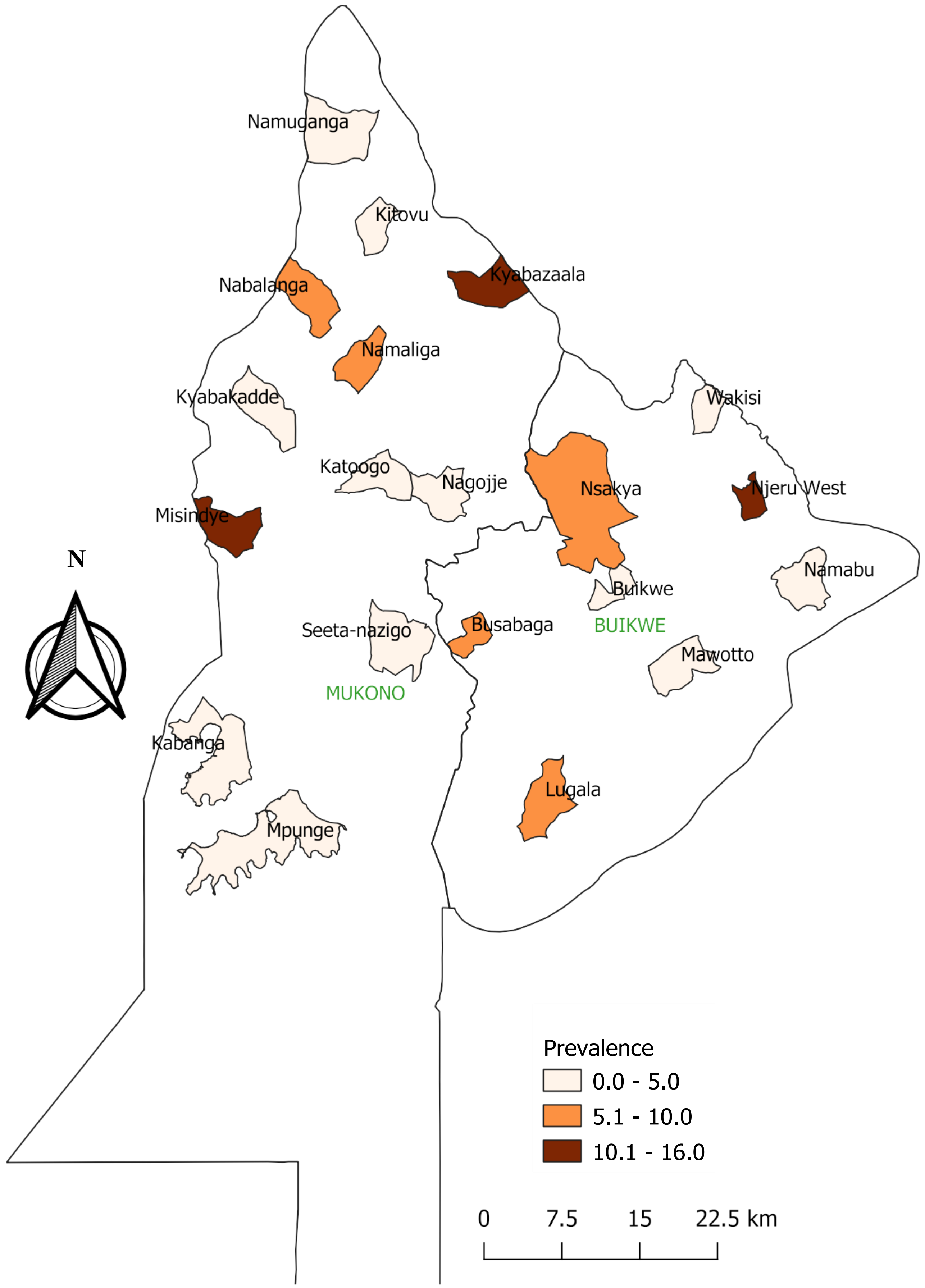

Map of Mukono and Buikwe districts showing the overall weighted prevalence of smoking by parish

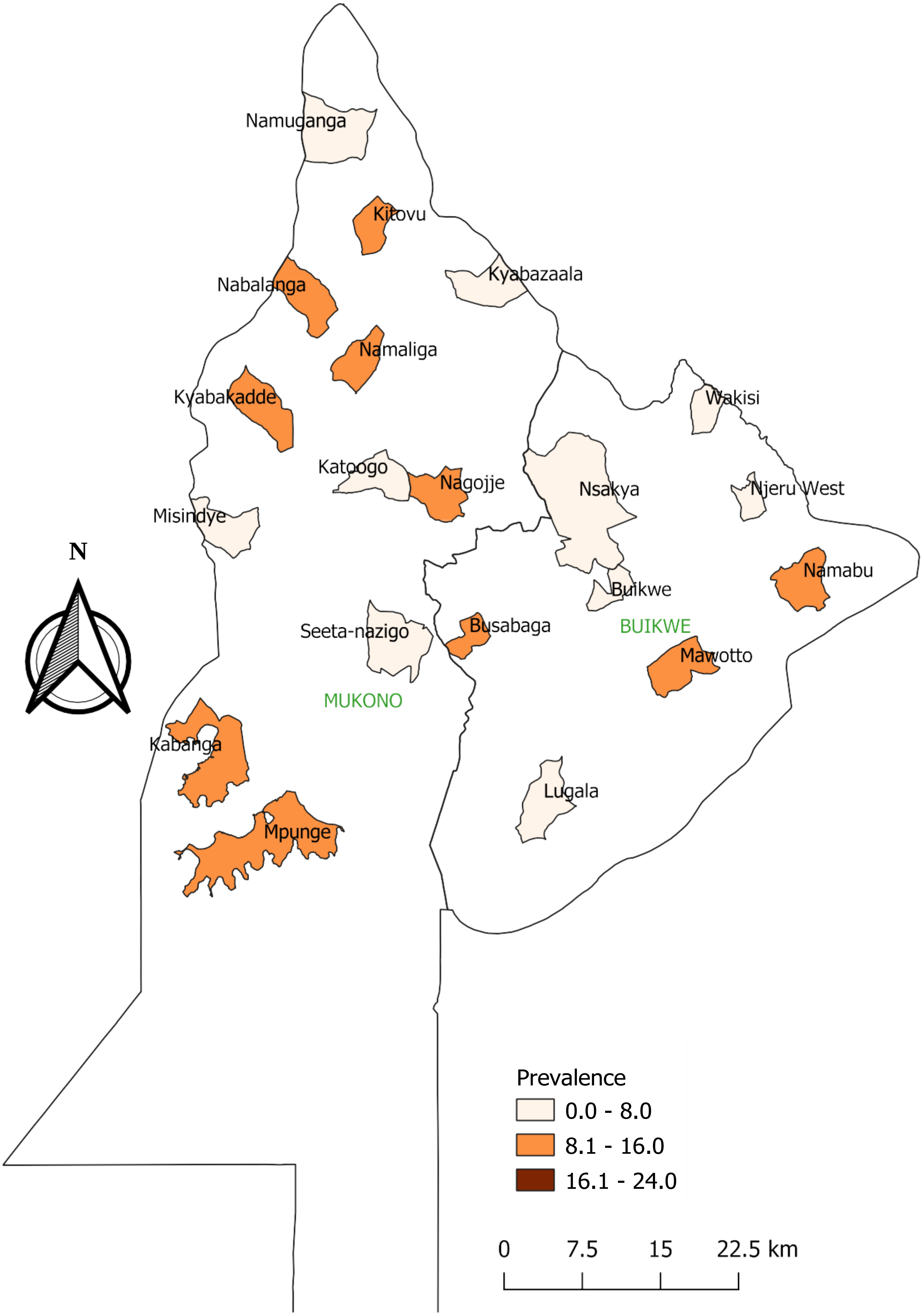

Map of Mukono and Buikwe districts showing the weighted prevalnce of smoking among men by parish

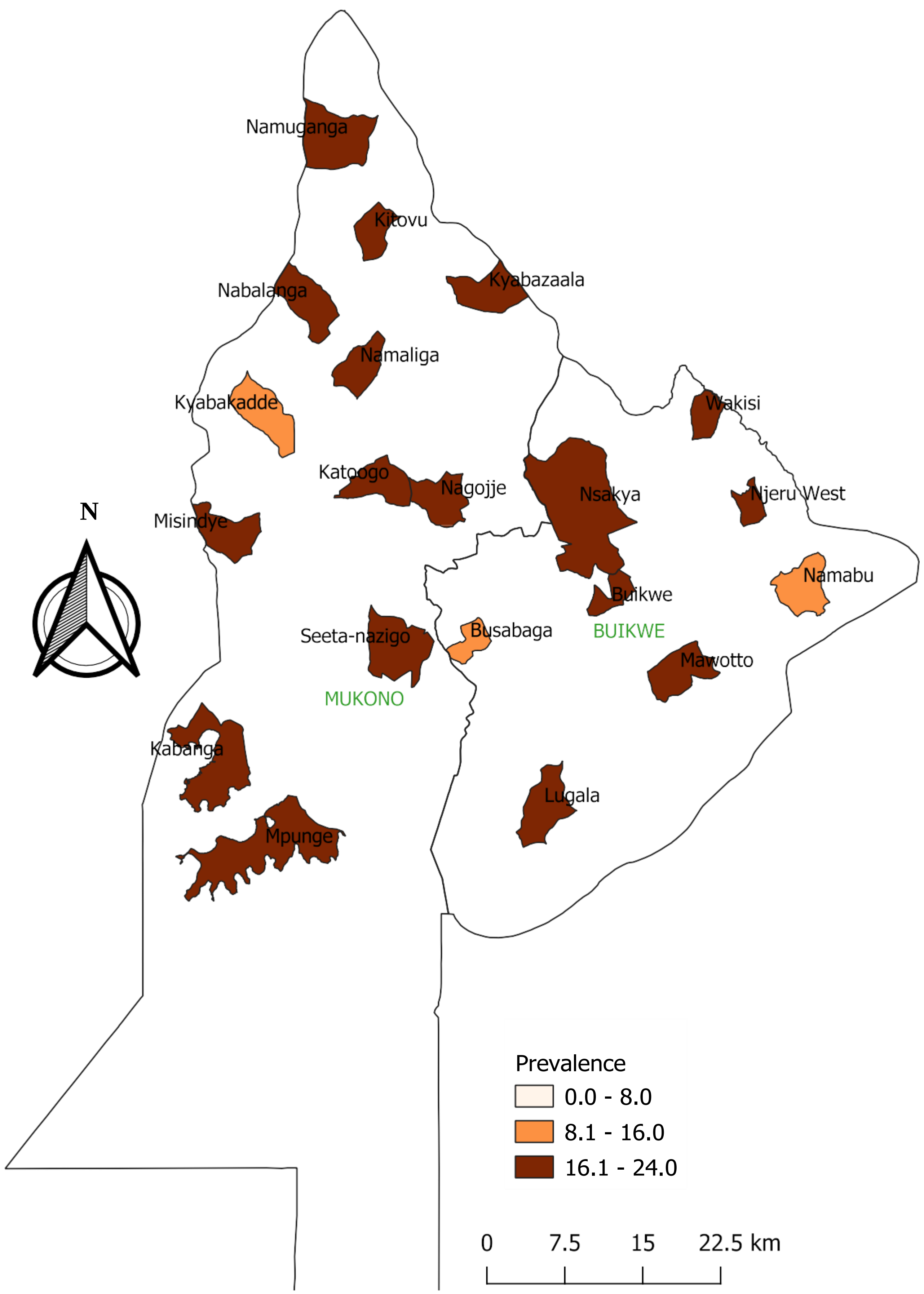

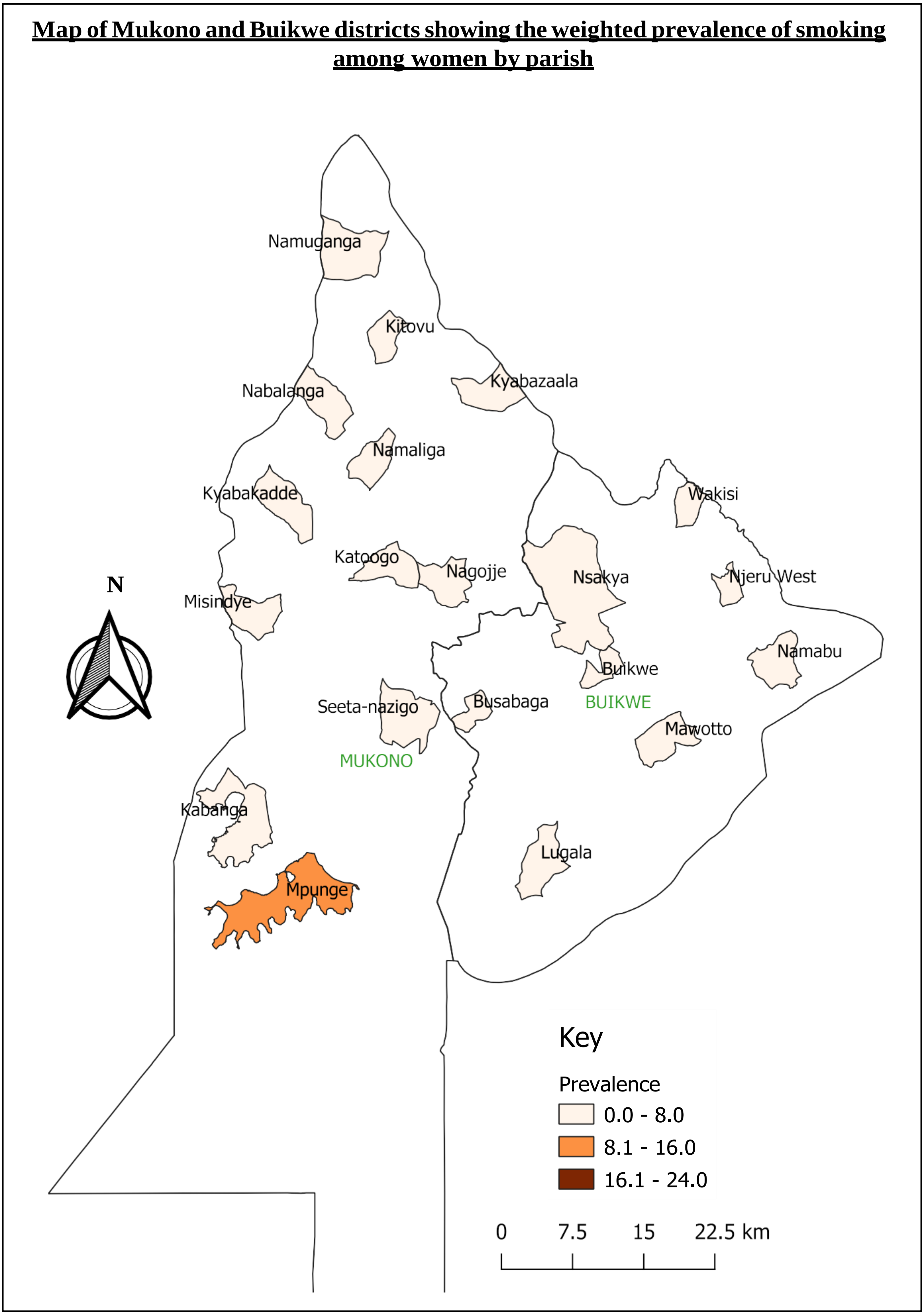
Map of Mukono and Buikwe districts showing the weighted prevalence of smoking among women by parish
N
Namuganga
Kitovu
Nabalanga
Kyabazaala
Namaliga
Kyabakadde
Wakisi
Katoogo
Nagojje
Nsakya
Njeru West
Misindye
Namabu
Buikwe
Seeta-nazigo
Busabaga
BUIKWE
Mawotto
MUKONO
Kabanga
Lugala
Mpunge
Key
Prevalence
0.0 - 8.0
8.1 - 16.0
16.1 - 24.0
0
7.5
15
22.5 km

Map of Mukono and Buikwe districts showing the overall weighted prevalence of alcohol consumption by parish

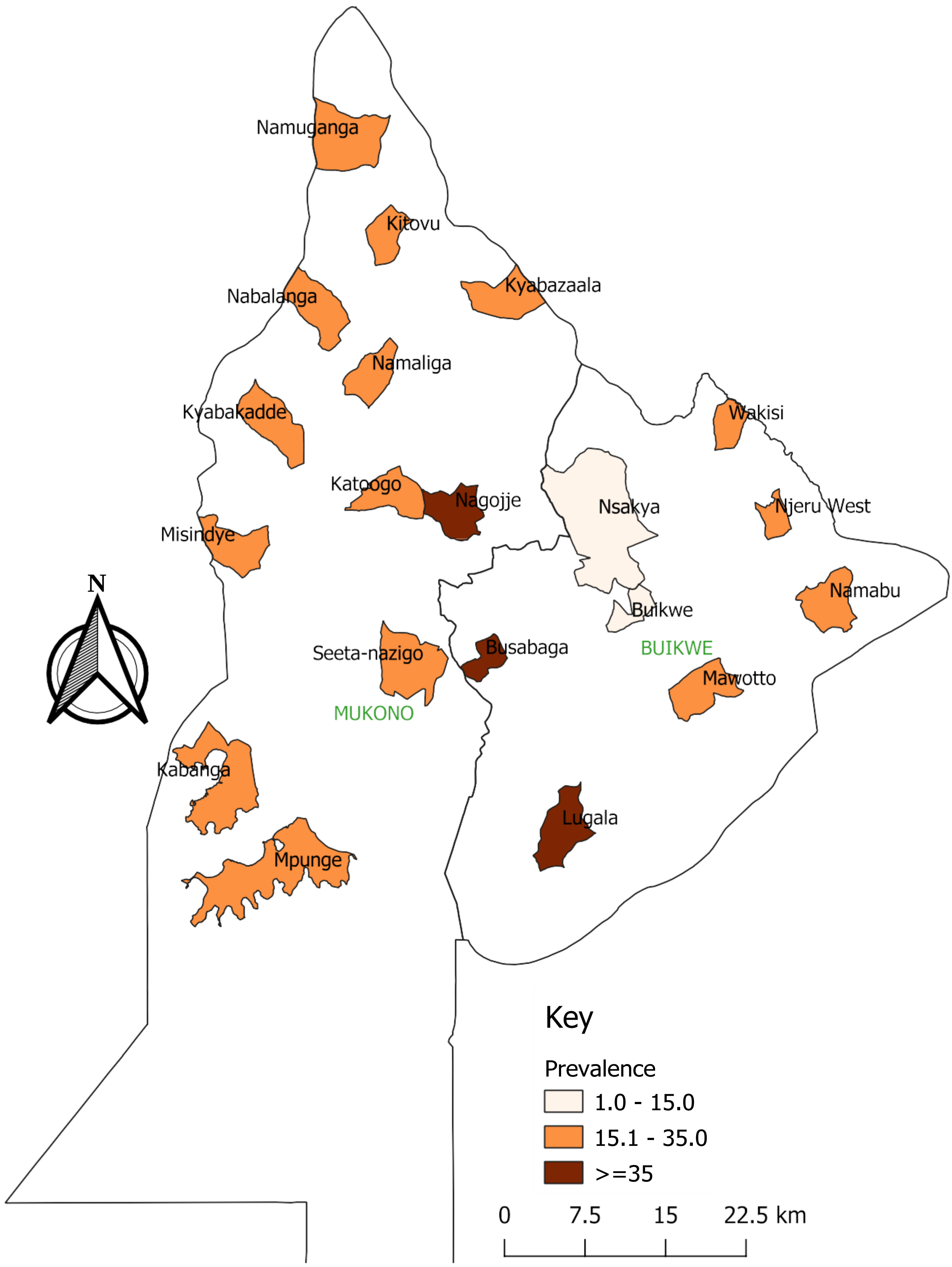

Map of Mukono and BUikwe districts showing the weighted prevalence of alcohol consumption among men by parish

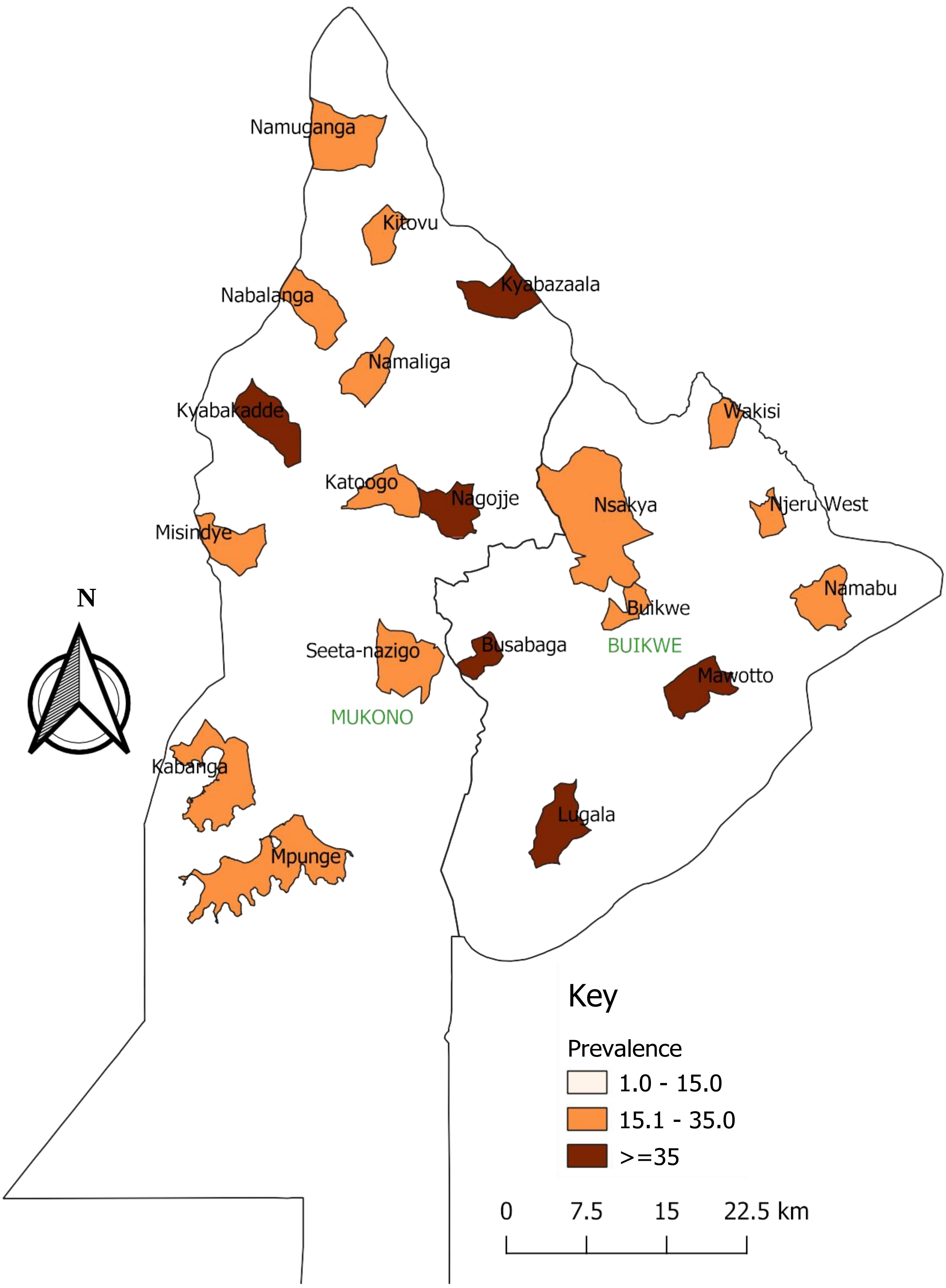

Map of Mukono and Buikwe districts showing the weighted prevalence of alcohol consumption among women by parish

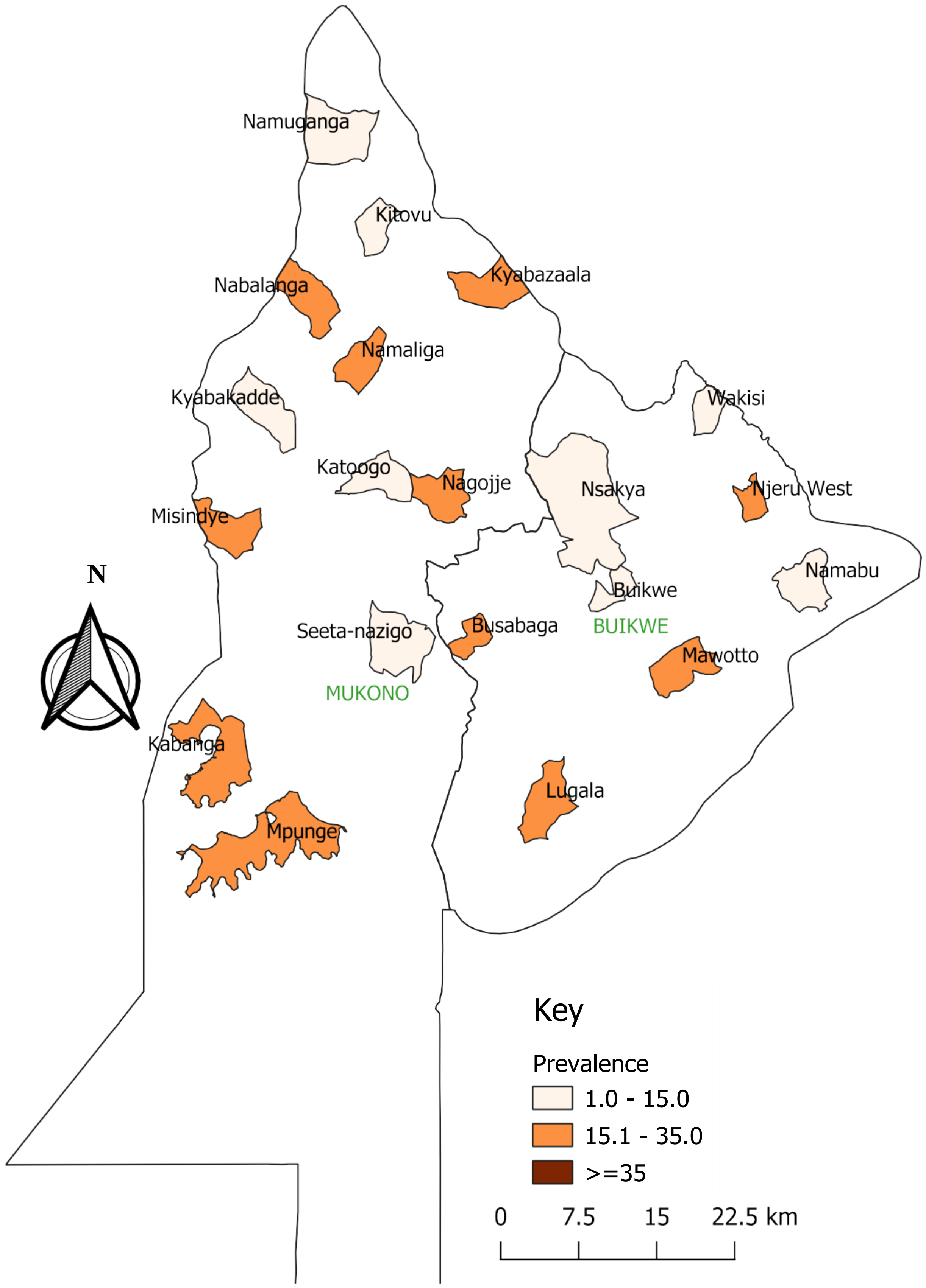

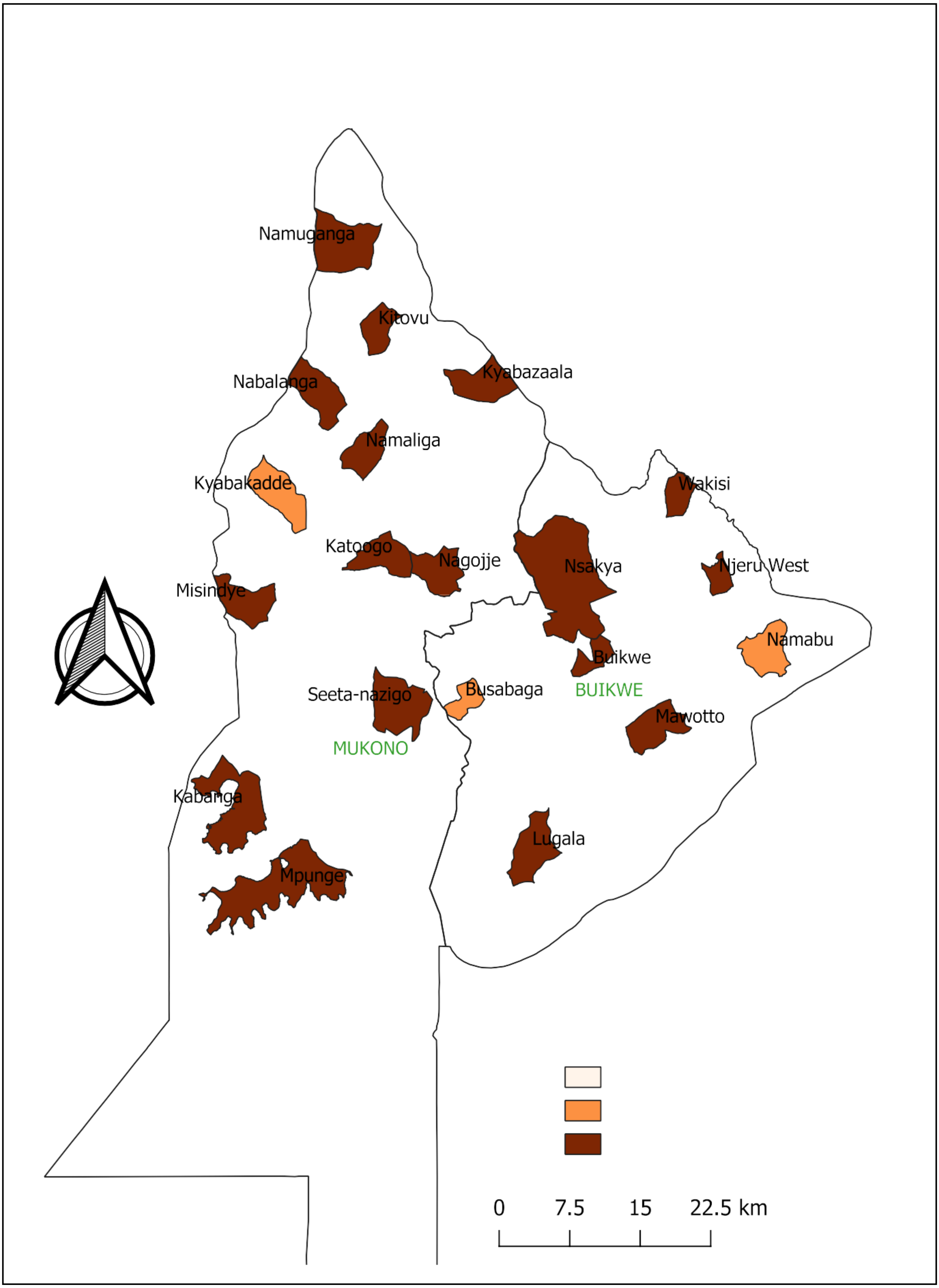
Namuganga
Kitovu
Nabalanga
Kyabazaala
Namaliga
Kyabakadde
Wakisi
Katoogo
Nagojje
Nsakya
Njeru West
Misindye
Namabu
Buikwe
Seeta-nazigo
Busabaga
BUIKWE
MUKONO
Mawotto
Kabanga
Lugala
Mpunge
0
7.5
15
22.5 km
